# Unsymmetrical Bisquinolines with High Potency against *P. falciparum* Malaria

**DOI:** 10.3390/molecules25092251

**Published:** 2020-05-10

**Authors:** Katherine M. Liebman, Steven J. Burgess, Bornface Gunsaru, Jane X. Kelly, Yuexin Li, Westin Morrill, Michael C. Liebman, David H. Peyton

**Affiliations:** 1 DesignMedix, Inc., Portland, OR 97201, USA; kliebman@pdx.edu (K.M.L.); sburgess@nogeire.com (S.J.B.); westin.morrill@gmail.com (W.M.); 2 Department of Chemistry, Portland State University, Portland, OR 97207, USA; bgunsaru@yahoo.com (B.G.); kellyja@ohsu.edu (J.X.K.); michael.liebman343@gmail.com (M.C.L.); 3 Portland VA Research Foundation, Portland, OR 97239, USA; liyu@ohsu.edu

**Keywords:** malaria, *P. falciparum*, drug discovery, SAR, structure–activity relationship, drug resistance

## Abstract

Quinoline-based scaffolds have been the mainstay of antimalarial drugs, including many artemisinin combination therapies (ACTs), over the history of modern drug development. Although much progress has been made in the search for novel antimalarial scaffolds, it may be that quinolines will remain useful, especially if very potent compounds from this class are discovered. We report here the results of a structure-activity relationship (SAR) study assessing potential unsymmetrical bisquinoline antiplasmodial drug candidates using in vitro activity against intact parasites in cell culture. Many unsymmetrical bisquinolines were found to be highly potent against both chloroquine-sensitive and chloroquine-resistant *Plasmodium falciparum* parasites. Further work to develop such compounds could focus on minimizing toxicities in order to find suitable candidates for clinical evaluation.

## 1. Introduction

Malaria remains a challenge for worldwide public health. The World Health Organization estimated the total number of deaths in 2018 to be 405,000, down from 585,000 in 2010 and 864,000 in 2000 [1,2]. While there has been considerable progress and optimism about malaria elimination, eradication may not be rapid. So as not to squander the progress that has been made, every possible new tool is needed, especially in light of the development of drug resistance to nearly every current therapeutic for *P. falciparum* malaria [3,4,5].

Bisquinolines have been explored for malaria for a long time, and the most successful of these, piperaquine (PPQ; Table 1), has been used as both mono- and combination therapy [6]. Piperaquine is a long-acting component of dihydroartemisinin-piperaquine, one of the artemisinin combination therapies recommended by the World Health Organization to treat *P. falciparum* malaria [7]. PPQ and a variety of analogs were first synthesized in France at Rhône-Poulenc [8,9,10,11] (Table 1) and further developed in China. Clinical trials performed in China during the early 1970s led to piperaquine’s wide use in China, both as treatment and as prophylaxis for *P. falciparum* and *P. vivax* malaria [12,13,14,15,16].

Piperaquine is believed to work by a mechanism similar to that of chloroquine and other 4-aminoquinolines [15]; however, it retains activity against many chloroquine-resistant (CQR) strains, perhaps because its large size prevents it being exported by the chloroquine resistance transporter [15,17,18,19,20]. However, following its introduction in China, resistance to PPQ monotherapy became common in the areas in which it was heavily used [12,13,14]. Unfortunately, resistance to the combination dihydroartemisinin–piperaquine has now arisen and continues to increase in southeast Asia [21,22,23]. Recent work is uncovering the mechanisms of piperaquine resistance [5,24,25,26,27,28,29].

In addition to PPQ itself, a wide range of other bis-4-aminoquinoline compounds have been shown to have antiplasmodial activity, tending to be active against both chloroquine-sensitive (CQS) and CQR strains [17,19,30,31,32,33,34,35,36,37,38,39,40]. During the 1990s, a study of bis-quinolines by Vennerstrom and co-workers led to the discovery of Ro 48–6910, which was evaluated in preclinical studies but was regrettably found to be phototoxic [19,37,41,42,43,44]. More recently, Kondaparla et al. have explored a series of unsymmetrical bisquinolines linked by an amide and an adjacent chiral center [17].

Here, we report on a series of simple but unsymmetrical bisquinolines based on the 4-aminoquinoline structure derived from CQ and other antimalarial drugs. One of these has unusually potent in vitro activity against CQS and CQR malaria, indicating that structures in this series may be worth further evaluation as antimalarials.

## 2. Results

In vitro antiplasmodial activities against *P. falciparum* are given in Table 1. The bisquinoline compounds vary in their alkylamine linkage between quinoline ring systems (alkyl, piperazine, or piperidine moieties). Compound **1** is similar to PPQ, except that it lacks the quinoline Cl atoms. Like piperaquine, the bisquinoline **5** was originally reported by Rhône-Poulenc during the 1960s [45]; it is here compared with a des-chloro analog, **7**. Finally, we report a series of compounds based on the unsymmetrical bisquinoline compound **6** that vary in their quinoline ring substitution pattern (7-chloro, 8-trifluoromethyl, or no substituent). For comparison to the bisquinolines, we also include compounds **2** and **4** from earlier work in our laboratory, together with **3,** the des-chloro analog of compound **2**.

As can be seen from these results, bisquinoline structures can give very potent antimalarial activities against the standard laboratory-adapted D6, Dd2, and 7G8 strains, the last two being accepted CQR strains. However, cytotoxicity of an unsymmetrical bisquinoline assessed in mouse spleen lymphocytes was found to be elevated relative to that of chloroquine.

**Table 1:** In vitro antiplasmodial activities of bisquinoline and related compounds.

Compound	Activity (IC_50_; nM)			Cytotoxicity	Structure
	D6	Dd2	7G8	(LC_50_; nM) Mouse Spleen Lymphocytes	
CQ	6.9	102	106	12,400	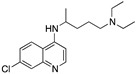
PPQ	0.7	1.5			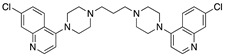
**1**	1.3	4.1	7.4		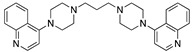
**2** [46]	2.4	3.7	1.5	1100	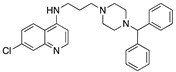
**3**	1.5	5.0		1600	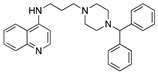
**4** (DM1157) [46,47]	0.2	2.2	1.8	6500	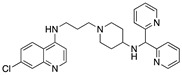
**5** [45]	0.4	0.7	0.1		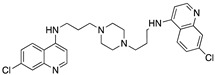
**6**	0.15	0.36	0.33	190	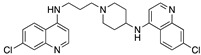
**7**	4.9	9.8	25		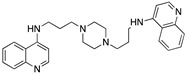
**8**	0.68	2.1	0.63		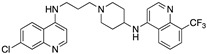
**9**	0.68	2.1	0.41		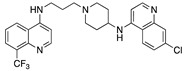
**10**	15	89	106		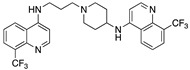
**11**	3.0	<2.5	<2.5		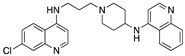
**12**	4.5	<2.5	<2.5		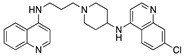
**13**	33	56	100		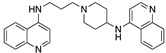

## 3. Discussion

**Figure 1:**
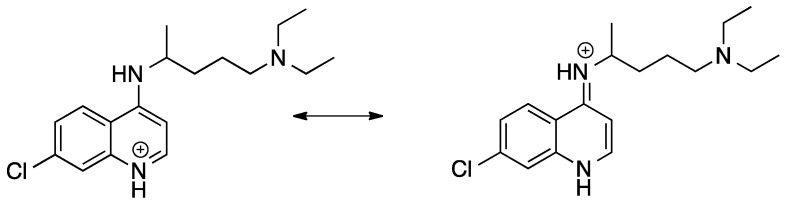
Resonance stabilization of the positive charge on the protonated ring nitrogen of chloroquine [55].

The in vitro antiplasmodial activities obtained for the bisquinolines reported here are given in Table 1, together with chloroquine, piperaquine, and three compounds from earlier work in our laboratory that contain a chloroquine moiety with an attached reversal agent (“reversed chloroquines”) [46,48]. Some of the compounds, particularly compound **6**, were found to be very active in vitro. Potency of these bisquinolines may be enhanced by the potential of both of the quinoline moieties in these molecules to interact with heme (a commonly invoked mechanism of action of the 4-aminoquinolines), an advantage that would also be available to piperaquine. However, a difference between compound **6** and piperaquine is that the 4-aminoquinoline nitrogens of compound **6** are secondary, while those of PPQ are tertiary; much of the earlier work on 4-aminoquinolines and 9-aminoacridines has focused on secondary 4-amino derivatives [49,50,51,52,53]. During the 1930s and 1940s, prior to research indicating that the site of action of 4-aminoquinolines and 9-aminoacridines is the parasite’s acidic digestive vacuole, Schönhofer and coworkers observed that alkylation of the 9-amino group of quinacrine (Atebrine) diminished antimalarial activity. To explain this, they proposed that antimalarial activity of quinacrine-like compounds required an ability to tautomerize, with the 9-NH shifting to the ring N (this is not possible for the tertiary 9-amino derivatives [49]). The in vitro antiplasmodial activity of chloroquine [15,50,54] as well as reversed chloroquine compounds (unpublished work from our laboratory) is also reduced, but not eliminated, by the presence of a tertiary 4-amino group. Egan has shown that aminoquinolines capable of stabilizing a positive charge on the quinoline ring nitrogen (2- and 4-aminoquinolines) have strong heme binding and are more basic, potentially leading to increased accumulation at the site of action in the parasite’s digestive vacuole (Figure 1 [55]).

While tertiary 4-aminoquinolines could also be capable of such resonance stabilization, it has been suggested that PPQ has steric hindrance between the proton in position 5 of the quinoline ring system and the piperazine methylene groups that results in twisting of the substituted 4-amino group out of the plane of the ring, reducing resonance stabilization of the alternative form. [15]. This would then result in reduced basicity of the ring nitrogen for such tertiary compounds, leading to reduced accumulation in the digestive vacuole. Experimental determination of the pK_a_s of piperaquine has shown that it is indeed less basic than CQ [15]. In spite of these observations, the bisquinoline PPQ–with two tertiary 4-aminoquinolines–is a highly active antimalarial. Warhurst proposed that while piperaquine’s accumulation in the aqueous acidic vacuole should be somewhat less than that of chloroquine, its accumulation in the lipid portions of the vacuole is expected to be much greater (based on the empirically determined pK_a_s and cLogP of both compounds; [15]). This may be significant in view of the evidence that hemozoin may form at the surface of or inside of lipid droplets within the digestive vacuole [15,56,57,58].

Whatever the reasons behind PPQ’s strong activity, the fact that compound **6** has even higher in vitro antiplasmodial potency over that of piperaquine may suggest that the presence of a tertiary amine is indeed detrimental to 4-aminoquinoline antimalarials and that piperaquine’s activity represents a compromise between the detriment of the tertiary 4-amino group and other more favorable properties. Compound **6** may possess the positive features of PPQ with the additional advantage of the secondary quinoline-4-amino group.

Bisquinoline compounds retain much of their activity against many CQR strains of *P. falciparum* malaria. To explain piperaquine’s activity against CQR strains, it has been suggested that the increased bulk and lipophilicity of PPQ relative to that of CQ causes the bisquinoline to be unable to be exported through the *P. falciparum* chloroquine resistance transporter (PfCRT) [15]. We have already suggested that reversed chloroquine molecules and PPQ can block export by PfCRT and so give high activity [59]. This may also apply to compound **6**, which is structurally similar to the reversed chloroquine compound **4** (DM1157) [46,47].

For the potent unsymmetrical bisquinoline compound **6** and its analogs, both ring systems seem to be of equal importance to the antiplasmodial activity. This conclusion is apparent from the various analogs with 8-trifluoromethyl substitution, which has been observed to be an inactivating substitution pattern for reversed chloroquine-type 4-aminoquinoline antiplasmodial candidates (unpublished work in our lab) and for other 4-aminoquinolines, including bisquinolines, although not for quinoline methanols, such as mefloquine [60,61,62,63,64] (and in one study, not for 4-aminobisquinolines where a 2-trifluoromethyl substituent was already present [65]). Thus, compound **10**, having the 8-CF_3_ substitution at both quinoline rings without the 7-Cl, loses at least two orders of magnitude of potency relative to compound **6**. However, for analogues of **6** with a single 8-trifluoromethyl substitution (compounds **8** and **9**), activity was almost as high as the parent **6**. These results suggest that either quinoline ring system is equally capable of participating in the antimalarial action of compound **6**.

Others have noted that the activity of CQ and its analogs is detrimentally affected by removal of the 7-chloro substituent [55,66,67,68,69,70,71,72,73], but we have observed that this may not be the case for reversed chloroquine compounds (see compounds **2** and **3**, Table 1; [48]). Here, we report that PPQ’s in vitro activity is minimally affected by removal of chlorine. For two other bisquinoline scaffolds, **5** [45] and **6**, we found that activity of des-chloro analogs was reduced compared to the 7-chloro parent (compare **6** to **11**, **12**, and **13** and **5** to **7**), although not as much as has been observed for chloroquine and analogs. Both **5** and **6** differ from piperaquine in having a secondary rather than tertiary 4-amino group. This result suggests that secondary amino bisquinolines are more sensitive to removal of chlorine than piperaquine. Even when just one ring system of **6** lacks a 7-chloro substituent, activity is slightly diminished relative to the parent **6**. For the 8-CF_3_ series, it appears that even with one ring system “inactivated”, antimalarial activity does not decrease significantly. This does not appear to be the case for des-chloro substitution.

## 4. Materials and Methods

Reagents and solvents were purchased from TCI America, Aldrich Chemical, Alfa Aesar, or Acros Organics and were used as received without further purification.

### 4.1. General Synthetic Methods

**Figure 2:**
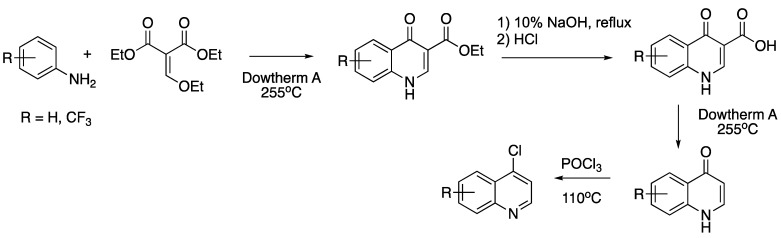
Synthesis of 4-chloroquinolines by the Gould–Jacobs reaction, followed by chlorination with phosphorus oxychloride.

**Figure 3:**
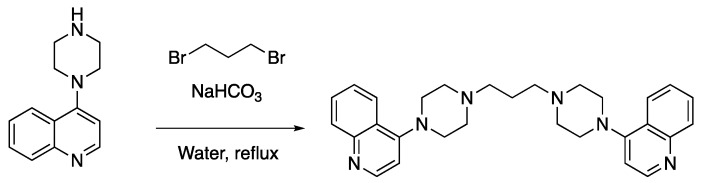
Synthesis of compound **1**.

**Figure 4:**
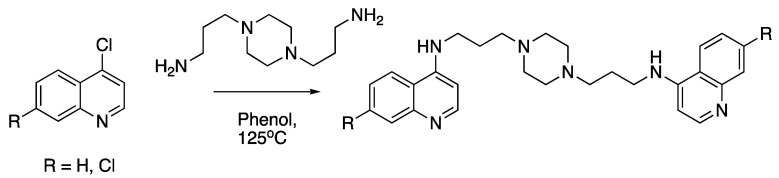
Synthesis of compounds **5** and **7**.

Except for 4,7-dichloroquinoline, substituted 4-chloroquinolines were made by the Gould–Jacobs reaction, followed by chlorination with phosphorus oxychloride (Figure 2 [74,75,76,77]):

Compound **1** was made by reaction of 4-(piperazin-1-yl)quinoline [78,79] with 1,3-dibromopropane (Figure 3):

Compound **5** [45] and **7** were made by the reaction of the appropriate 4-chloroquinoline with 1,4-bis(3-aminopropyl)piperazine in phenol (Figure 4 [51,80]):

The synthesis of the unsymmetrical bisquinoline compounds required the synthesis of two different 4-aminoquinoline starting materials: a 3-(quinolin-4-ylamino)propyl methanesulfonate and an N-(piperidin-4-yl)quinolin-4-amine.

**Figure 5:**
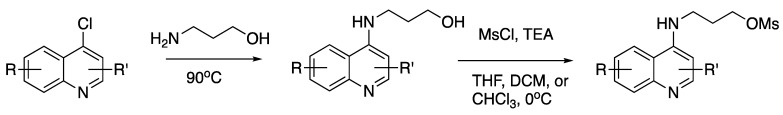
Reaction of 4-chloroquinolines with 3-amino-1-propanol, followed by activation of the resulting alcohol by methanesulfonyl chloride.

**Figure 6:**
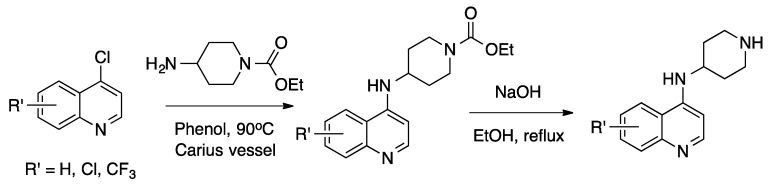
Synthesis of one starting material for unsymmetrical bisquinoline compounds.

**Figure 7:**
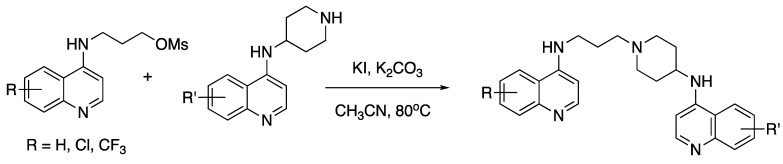
Synthesis of unsymmetrical bisquinoline compounds.

To make the 3-(quinolin-4-ylamino)propyl methanesulfonates, the appropriate 4-chloroquinoline was allowed to undergo solvolysis with 3-amino-1-propanol to give a 4-amino alcohol, and the hydroxyl group was then converted to a methanesulfonyl group (Figure 5 [46,59]):

The *N*-(piperidin-4-yl)quinolin-4-amines were synthesized by reaction of the appropriate 4-chloroquinoline with 1-carbethoxy-4-aminopiperidine in phenol [51,80], followed by removal of the carbethoxy group by heating with aqueous caustic soda in ethanol (Figure 6 [81]):

Reaction of the two quinoline starting materials then provided the desired bisquinolines (Figure 7 [46,82]):

Bisquinoline products were purified by recrystallization and sometimes column chromatography. A different, less satisfactory synthetic method was initially used to obtain compound **6**. This is detailed in the Supplementary Materials.

### 4.2. Characterization of Products

Bisquinoline final products were characterized by NMR (^1^H, ^13^C, COSY, HSQC, NOESY, and ^19^F if applicable), HPLC, and HR-MS (details of methods used below). Intermediates were characterized by ^1^H NMR and in some cases HPLC and HR-MS.

^1^H, ^13^C, COSY, HSQC, HMBC, and ^19^F NMR experiments were run on a Bruker 400 MHz or 600 MHz spectrometer. (Note: Trifluoromethyl carbons were not observed in ^13^C NMR spectra, likely due to reduction in intensity due to ^19^F–^13^C splitting as well as the lack of the 1-bond ^1^H–^13^C nuclear Overhauser effect (NOE).) HPLC was performed using UV detection at 254 and 325 nm using a Varian ProStar 325 UV/vis dual wavelength detector. Three HPLC methods were used. For HPLC method A, a SUPELCO Ascentis RP-Amide 5 μm, 4.6 mm × 150 mm column was used, eluting with a gradient of 95:5 to 30:70 water with 0.1% formic acid (*v*/*v*)/acetonitrile. For HPLC method B, a SUPELCO Ascentis C18 5 μm, 4.6 × 150 mm column was used, eluting with 95% water and 5% acetonitrile. For HPLC method C, a SUPELCO Ascentis C18 5 μm, 4.6 × 150 mm column was used, eluting with a gradient from 95:5 to 5:95 water with 0.1% formic acid (*v*/*v*)/acetonitrile. High resolution mass spectrometry was performed on a Bruker micrOTOF-Q instrument. Results were obtained using electrospray ionization (ESI) in the positive mode at a flow rate of 0.4 mL/min with 1:1 methanol–water. Gas chromatography–mass spectrometry (GC–MS) was performed using a Hewlett Packard HP5890 Series II gas chromatograph with a 30-m DB5 column. The oven temperature was set at 130 °C for 2 min and then increased to 300 °C at the rate of 30 °C per minute. This instrument was used with the kind permission of Dr. Michael Riscoe of the Portland Veterans Affairs Medical Center.

#### Example Synthesis: Compound **6**

(Further synthetic methods are provided in the Supplementary Materials).

**Figure 8:**
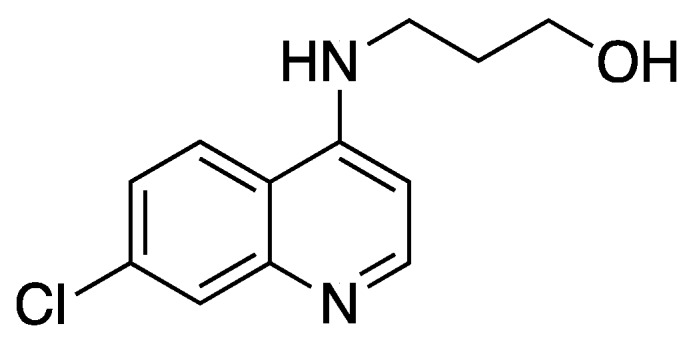
Structure of 3-(7-Dichloroquinolin-4-ylamino)propanol.

3-(7-Dichloroquinolin-4-ylamino)propanol (Figure 8 [59,82]):

The title compound was synthesized without deviating from methods previously described [59,82] (a pale tan solid, mp = 148.5–151.0 °C).

**Figure 9:**
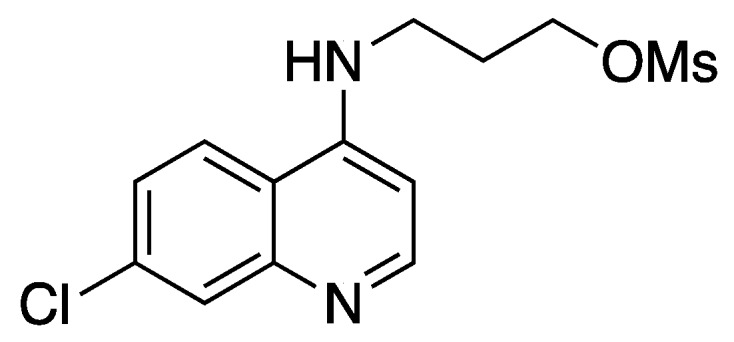
Structure of 3-(7-Chloroquinolin-4-ylamino)propyl methanesulfonate.

3-(7-Chloroquinolin-4-ylamino)propyl methanesulfonate (Figure 9 [59,82]):

3-(7-Dichloroquinolin-4-ylamino)propanol (1.88 g, 7.9 mmol), triethylamine (1.66 mL, 1.2 mmol), and anhydrous THF (100 mL) were cooled below 0 °C on ice/salt, and methanesulfonyl chloride (0.71 mL, 9.1 mmol) was added dropwise. After stirring for an hour on ice, TLC indicated that reaction was not complete, and therefore additional triethylamine (0.83 mL, 6.0 mmol) and methanesulfonyl chloride (0.36 mL, 6.0 mmol) were added. After a further hour, TLC indicated that no quinoline starting material remained. The reaction mixture was diluted with ethyl acetate (30 mL) and shaken with saturated sodium bicarbonate (30 mL), followed by extraction of the aqueous layer with additional ethyl acetate (3 × 10 mL). The pooled ethyl acetate layers were washed with brine (10 mL), dried over magnesium sulfate, and evaporated under reduced pressure with warming to obtain a pale yellow, fluffy solid (1.86 g, 81%).

^1^H NMR δ (ppm)(CDCl_3_): 8.53 (1 H, d, *J* = 5.37 Hz, ClQ-C2-H), 7.95 (1 H, d, *J* = 2.18 Hz, ClQ-C8-H), 7.72 (1 H, d, *J* = 8.97 Hz, ClQ-C5-H), 7.38 (1 H, dd, *J* = 8.94, 2.18 Hz, ClQ-C6-H), 6.42 (1 H, d, *J* = 5.40 Hz, ClQ-C3-H), 5.55 (1 H, br t, *J* = 5.75 Hz, NH), 4.42 (2 H, t, *J* = 5.66 Hz, CH_2_O), 3.58 (2 H, td, *J* = 6.34, 5.77 Hz, CH_2_N), 3.06 (3 H, s, CH_3_), 2.18 (2 H, m, CH_2_).

**Figure 10:**
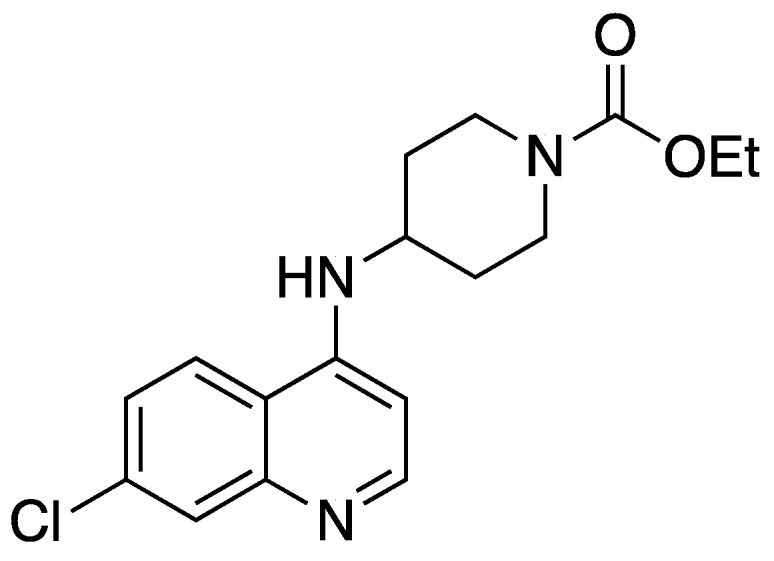
Structure of Ethyl 4-((7-chloroquinolin-4-yl)amino)piperidine-1-carboxylate.

Ethyl 4-((7-chloroquinolin-4-yl)amino)piperidine-1-carboxylate (Figure 10 [51,80])

4,7-Dichloroquinoline (2.00 g, 10 mmol), ethyl 4-amino-1-piperidine carboxylate (1.83 g, 11 mmol), and phenol (5.70 g, 61 mol) were heated at 90 °C in a sealed Carius vessel for 48 h. TLC indicated that unreacted 4,7-dichloroquinoline remained, and therefore additional ethyl 4-amino-1-piperidine carboxylate (0.39 g, 2.3 mmol) was added. The vessel was again sealed and heated for a further 7 days, whereupon TLC indicated that no unreacted quinoline remained. The reaction mixture was diluted with chloroform (50 mL) and rinsed with 10% caustic soda (6 × 10 mL), followed by further rinsing with brine (3 × 10 mL). The organic layer was dried over MgSO_4_ and concentrated under reduced pressure with warming to yield a thick, tan liquid containing some solid material. After standing 14 h, this was taken up in boiling solvent (50/50 ethyl acetate/95% ethanol (*v*/*v*)) and allowed to cool and concentrate at room temperature. The crystals thus formed were recovered from the remaining 5 mL of solvent by vacuum filtration (off-white crystals, 1.18 g, 35%, mp = 197.3–198.8 °C).

^1^H NMR δ (ppm)(CDCl_3_): 8.54 (1 H, d, *J* = 5.36 Hz), 7.96 (1 H, d, *J* = 2.17 Hz), 7.66 (1 H, d, *J* = 8.98 Hz), 7.37 (1 H, dd, *J* = 8.94, 2.19 Hz), 6.46 (1 H, d, *J* = 5.41 Hz), 4.92 (1 H, br d, *J* = 7.25 Hz), 4.16 (4 H, br s overlaps q, *J* = 7.13 Hz), 3.68–3.69 (1 H, m), 3.04 (2 H, td, *J* = 12.56, 2.82 Hz), 2.11–2.20 (2 H, m), 1.51–1.53 (2 H, m), 1.28 (3 H, t, *J* = 7.11 Hz).

MS (ESI): *m/z* 334.13271 M + H (calculated 334.13168).

HPLC (method A) *t*_R_ = 10.55 min (99% pure).

**Figure 11:**
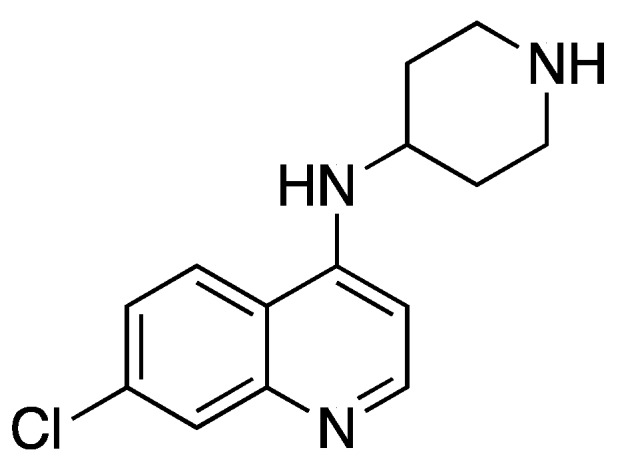
Structure of 7-Chloro-*N*-(piperidin-4-yl)quinolin-4-amine.

7-Chloro-*N*-(piperidin-4-yl)quinolin-4-amine (Figure 11 [81])

Ethyl 4-((7-chloroquinolin-4-yl)amino)piperidine-1-carboxylate (2.45 g, 7.3 mmol), 95% ethanol (100 mL), and 10% caustic soda (4.5 mL) were allowed to heat, stirring, at reflux for 4 days. As TLC indicated that the reaction was not complete, 0.5 mL of 50% caustic soda was added, and reflux was continued for a further 3 days. TLC then indicated that the reaction was complete. The reaction solvent was removed under reduced pressure with warming, and the residue was partitioned between chloroform (20 mL) and water (50 mL). After separation, the aqueous layer was extracted with additional chloroform (3 × 10 mL), and the pooled organic layers were dried (MgSO_4_) and concentrated under reduced pressure with warming to yield a tan solid (1.05 g). A cream-colored solid was also isolated from the aqueous layer by vacuum filtration (1.04 g). NMR indicated that both solids obtained were the desired product (total yield 1.83 g, 96%, mp = 166.3–169.4 °C).

^1^H NMR δ (ppm)(CDCl_3_): 8.52 (1 H, d, *J* = 5.39 Hz), 7.96 (1 H, d, *J* = 2.18 Hz), 7.65 (1 H, d, *J* = 8.96 Hz), 7.37 (1 H, dd, *J* = 8.93, 2.19 Hz), 6.45 (1 H, d, *J* = 5.42 Hz), 4.87 (1 H, br d, *J* = 7.35 Hz), 3.61–3.62 (1 H, m), 3.19 (2 H, dt, *J* = 12.68, 3.70 Hz), 2.79–2.81 (2 H, m), 2.15–2.19 (2 H, m), 1.50–1.50 (2 H, m).

MS (ESI): *m/z* 262.11116 M + H (calculated 262.11065).

HPLC (method A) *t*_R_ = 2.74 min (94% pure).

**Figure 12:**
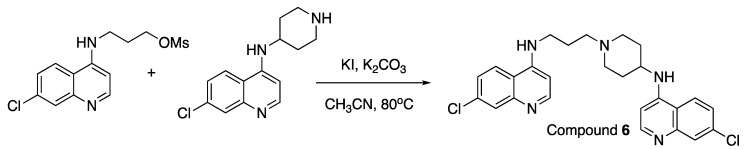
Synthesis of Compound **6**.

Compound **6** (7-Chloro-*N*-(3-(4-((7-chloroquinolin-4-yl)amino)piperidin-1-yl)propyl)quinolin-4-amine) (Figure 12 [46,82])

3-(7-Chloroquinolin-4-ylamino)propyl methanesulfonate (1.20 g, 3.8 mmol), 7-chloro-*N*-(piperidin-4-yl)quinolin-4-amine (1.05 g, 4.0 mmol), potassium carbonate (5.7 mmol, 0.79 g), a catalytic amount of potassium iodide, and 50 mL anhydrous acetonitrile were allowed to heat for 48 h at reflux, whereupon TLC indicated that the reaction was complete. The reaction mixture was diluted with water (50 mL) and vacuum filtered. The filtrate was concentrated under reduced pressure with warming, and the reaction mixture was partitioned between 50/50 dichloromethane/chloroform (20 mL) and 10 mL saturated sodium bicarbonate, followed by further extraction with three 10 mL portions of dichloromethane. The pooled organic layers were dried over anhydrous magnesium sulfate and concentrated under reduced pressure. The resulting solid was combined with the material filtered from the reaction mixture and recrystallized from 95% ethanol, which afforded the desired product as a pale yellow, crystalline solid (1.30 g). Concentration of the mother liquor yielded a further crop of crystals (0.06 g, total yield 71%, mp = 224–227 °C (dec)).

^1^H NMR δ (ppm)(CDCl_3_): 8.56 (1 H, d, *J* = 5.33 Hz, Q_1_-C2-H), 8.53 (1 H, d, *J* = 5.33 Hz, Q_2_-C2-H), 7.99 (1 H, d, *J* = 2.16 Hz, Q_1_-C5-H), 7.97 (1 H, d, *J* = 2.14 Hz, Q_2_-C5-H), 7.78 (1 H, d, *J* = 8.90 Hz, Q_2_-C8-H), 7.71 (1 H, d, *J* = 8.95 Hz, Q_1_-C8-H), 7.43 (1 H, dd, *J* = 8.89, 2.18 Hz, Q_1_-C6-H), 7.35 (1 H, dd, *J* = 8.87, 2.16 Hz, Q_2_-C6-H), 7.02 (1 H, br t, *J* = 4.27 Hz, Q_1_-C4-NH), 6.47 (1 H, d, *J* = 5.37 Hz, Q_1_-C3-H), 6.38 (1 H, d, *J* = 5.37 Hz, Q_2_-C3-H), 4.92 (1 H, br d, *J* = 6.78 Hz, Q_2_-NH), 3.65 (1 H, m, Pip-CH), 3.42 (2 H, td, J_CH2_ = 6.01, J_NH_ = 4.35 Hz, Q_1_-NHCH_2_CH_2_CH_2_), 3.06 (2 H, m, piperidine-CH × 2 adjacent to alkyl chain), 2.67 (2 H, t, J_CH2_ = 5.64 Hz, Q_1_-NHCH_2_CH_2_CH_2_), 2.33 (2 H, m, piperidine-CH × 2 adjacent to alkyl chain), 2.28 (2 H, m, piperidine CH × 2 adjacent to CH-NH-Q_2_), 1.99 (2 H, m, Q_1_-NHCH_2_CH_2_CH_2_), 1.75 (water signal overlaps m, ~2 H, piperidine CH × 2 adjacent to CH-NH-Q_2_).

^13^C NMR δ (ppm)(CDCl_3_): 152.3 (Q_1_-C2), 152.0 (Q_2_-C2), 150.4, 149.4, 149.3, 148.4, 135.1, 134.7, 129.1 (Q-C5), 129.0 (Q-C5), 125.6 (Q_1_-C6), 124.8 (Q_2_-C6), 121.7 (Q_2_-C8), 120.7 (Q_1_-C8), 117.5, 117.2, 99.6 (Q_1_-C3), 98.8 (Q_2_-C3), 58.2 (Q_1_-NHCH_2_CH_2_CH_2_), 52.5 (piperidine-C adjacent to alkyl chain), 49.5 (piperidine-CH-NH-Q_2_), 43.9 (Q_1_-NHCH_2_CH_2_CH_2_), 32.0 (piperidine-C adjacent to CH-NH-Q_2_), 24.4 (Q_1_-NHCH_2_CH_2_CH_2_).

Note: Q_1_ and Q_2_ denote the quinoline ring system on the left and that on the right of the structure, respectively, as shown above. Spectra are provided in Supplementary Materials (Example spectra: Compound **6**).

MS (ESI): *m/z* 480.17456 M + H (calculated 480.17163).

HPLC (method A) *t*_R_ = 6.93 min (97% pure).

### 4.3. In Vitro Studies on Inhibition of P. falciparum Parasite Growth

The antiplasmodial activities of the compounds in this study were determined by methods described previously [46,83]. The following three strains of *P. falciparum* were used: (1) a chloroquine-sensitive strain, D6; (2) a chloroquine-resistant strain, Dd2, originally isolated from southeast Asia; and (3) a second chloroquine-resistant strain, 7G8, originally isolated from Brazil. The parasites were maintained continuously in culture, and asynchronous cultures were used for testing. Samples of the cultures were diluted to 0.2% parasitemia and 0.2% hemocrit using uninfected red blood cells and complete cell growth medium (RPMI-1640 with 0.5% Albumax II). Chloroquine was used as a positive control. Solutions of chloroquine and the test compounds were made at 10 mM in DMSO. These solutions were diluted into complete cell growth medium. In a 96-well microplate, the stock solutions were diluted with complete cell growth medium to provide triplicate wells at concentrations between 0 and 10^−4^ M, each having a final volume of 100 μL. A given assay was performed using concentrations either in the range of 0.025 to 250 nM or 2.5 to 2500 nM. The plates were then incubated under standard culture conditions for 72 h before harvesting. The SYBR Green-I fluorescence-based method [83] was used to read the plates using a 96-well plate fluorescence reader (Gemini-EM, Molecular Devices) with excitation and emission wavelengths of 497 and 520 nm, respectively. Fluorescence was plotted against the logarithm of drug concentration. IC_50_ values were then obtained by curve fitting by nonlinear regression analysis using Prism (Graph Pad) software. The IC_50_ obtained for the chloroquine-positive control was then used to “normalize” the IC_50_ values obtained for the test compounds to the chloroquine IC_50_ values of 6.9 nM^D6^, 102 nM^Dd2^, and 108 nM^7G8^ [46].

## 5. Conclusions

The high in vitro antiplasmodial activity of compound **6** and its synthetic accessibility makes this a compound of interest for further development as a potential antimalarial drug, particularly if it were found to be active against piperaquine-resistant strains of *Plasmodium*, which is the case (D. Fidock, private communication). However, for bisquinolines as well as for other classes of compounds, high in vitro activity does not necessarily predict high activity in vivo. Additionally, the substantially elevated cytotoxicity of **6** relative to CQ are cause for possible concern. It may be possible to improve this feature by the design of further analogs within this series.

## Supplementary Materials

The following are available online, Synthetic details for all compounds not described in the main manuscript; Figure C1–C12: Detailed ^1^H & ^13^C NMR spectra for Compound **6**.
