# Supplementary material for: Unsymmetrical Bisquinolines with High Potency against P. falciparum Malaria
Source: Molecules. 2020 May 10;25(9):2251. doi: 10.3390/molecules25092251 (PMC7249153; doi:10.3390/molecules25092251)
Supplement: Supplementary file 1 [file molecules-25-02251-s001.pdf]

SUPPLEMENTARY MATERIALS for:

Unsymmetrical Bisquinolines with High Potency Against *P. falciparum* malaria

Katherine M. Liebman [1,2], Steven J. Burgess [1], Bornface Gunsaru [2], Jane X. Kelly [2,3], Yuexin Li [3], Westin Morrill [1], Michael C. Liebman [2] and David H. Peyton [1,2*]

[1] DesignMedix, Inc., Portland, Oregon, USA
[2] Department of Chemistry, Portland State University, Portland, Oregon, USA
[3] Portland VA Research Foundation, Portland, Oregon, USA
* Correspondence: peytond@pdx.edu; Tel.: +01-503-805-1291

Note: Trifluoromethyl carbons were not observed in ^{13}C NMR spectra, likely due to reduction in intensity due to ^{19}F-^{13}C splitting, as well as the lack of the 1-bond ^{1}H-^{13}C nuclear Overhauser effect (NOE).

3-(7-Dichloroquinolin-4-ylamino)propanol (Burgess, 2006; Burgess, 2010)

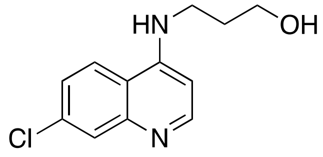

The title compound was synthesized without deviating from methods previously described (Burgess, 2006; a pale tan solid, mp = 148.5-151.0°C).

3-(7-Chloroquinolin-4-ylamino)propyl methanesulfonate (Burgess, 2006; Burgess, 2010; Andrews, 2010):

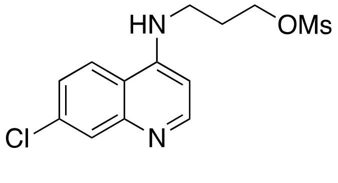

3-(7-Dichloroquinolin-4-ylamino)propanol (1.88 g, 7.9 mmol), triethylamine (1.66 mL, 1.2 mmol), and anhydrous THF (100 mL) were cooled below 0°C on ice/salt, and methanesulfonyl chloride (0.71 mL, 9.1 mmol) was added dropwise. After stirring for an hour on ice, TLC indicated that reaction was not complete, and therefore additional

triethylamine (0.83 mL, 6.0 mmol) and methanesulfonyl chloride (0.36 mL, 6.0 mmol) were added. After a further hour, TLC indicated that no quinoline starting material remained. The reaction mixture was diluted with ethyl acetate (30 mL) and shaken with saturated sodium bicarbonate (30 mL), followed by extraction of the aqueous layer with additional ethyl acetate (3 x 10 mL). The pooled ethyl acetate layers were washed with brine (10 mL), dried over magnesium sulfate, and evaporated under reduced pressure with warming to obtain a pale yellow, fluffy solid; this was allowed to stand overnight (1.86 g, 81%).

^{1}H NMR δ (ppm)($CDCl_3$): 8.53 (1 H, d, J = 5.37 Hz, ClQ-C2-H), 7.95 (1 H, d, J = 2.18 Hz, ClQ-C8-H), 7.72 (1 H, d, J = 8.97 Hz, ClQ-C5-H), 7.38 (1 H, dd, J = 8.94, 2.18 Hz, ClQ-C6-H), 6.42 (1 H, d, J = 5.40 Hz, ClQ-C3-H), 5.55 (1 H, br t, J = 5.75 Hz, NH), 4.42 (2 H, t, J = 5.66 Hz, CH_2O), 3.58 (2 H, td, J = 6.34, 5.77 Hz, CH_2N), 3.06 (3 H, s, CH_3), 2.18 (2 H, m, CH_2).

^{1}H NMR δ (ppm)(DMSO-d_6): 8.42 (1 H, d, J = 5.39 Hz, ClQ-C2-H), 8.26 (1 H, d, J = 9.04 Hz, ClQ-C5-H), 7.79 (1 H, d, J = 2.24 Hz, ClQ-C8-H), 7.47 (1 H, dd, J = 8.98, 2.27 Hz, ClQ-C6-H), 7.33 (1 H, br t, J = 5.39 Hz, Q-C4-NH), 6.51 (1 H, d, J = 5.45 Hz, ClQ-C3-H), 4.35 (1 H, t, J = 6.19 Hz, $HNCH_2CH_2C\underline{H}_2$), 3.30-3.45 (water signal overlaps m, ~1 H, $HNC\underline{H}_2CH_2CH_2$), 3.19 (1 H, s, CH_3), 2.07-2.09 (1 H, m, $HNCH_2C\underline{H}_2CH_2$).

Ethyl 4-((7-chloroquinolin-4-yl)amino)piperidine-1-carboxylate (Andersag, 1948; Surrey, 1951)

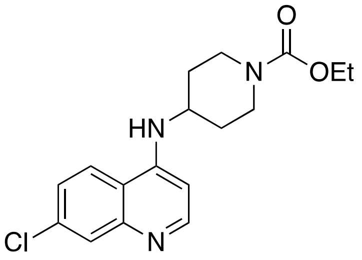

4,7-Dichloroquinoline (2.00 g, 10 mmol), ethyl 4-amino-1-piperidine carboxylate (1.83 g, 11 mmol), and phenol (5.70 g, 61 mol) were heated at 90ºC in a sealed Carius vessel for 48 hours. TLC indicated that unreacted 4,7-dichloroquinoline remained, and therefore additional ethyl 4-amino-1-piperidine carboxylate (0.39 g, 2.3 mmol) was added. The vessel was again sealed and heated for a further 7 days, whereupon TLC indicated that no unreacted quinoline remained. The reaction mixture was diluted with chloroform (50 mL) and rinsed with 10% caustic soda (6 x 10 mL), followed by further rinsing with brine (3 x 10 mL). The organic layer was dried over $MgSO_4$ and concentrated under

reduced pressure with warming to yield a thick, tan liquid containing some solid material. After standing 14 hours, this was taken up in boiling solvent (50/50 ethyl acetate/95% ethanol (v/v)) and allowed to cool and concentrate at room temperature. The crystals thus formed were recovered from the remaining 5 mL of solvent by vacuum filtration (off-white crystals, 1.18 g, 35%, mp = 197.3-198.8°C).

^{1}H NMR δ (ppm)($CDCl_3$): 8.54 (1 H, d, J = 5.36 Hz), 7.96 (1 H, d, J = 2.17 Hz), 7.66 (1 H, d, J = 8.98 Hz), 7.37 (1 H, dd, J = 8.94, 2.19 Hz), 6.46 (1 H, d, J = 5.41 Hz), 4.92 (1 H, br d, J = 7.25 Hz), 4.16 (4 H, br s overlaps q, J = 7.13 Hz), 3.68-3.69 (1 H, m), 3.04 (2 H, td, J = 12.56, 2.82 Hz), 2.11-2.20 (2 H, m), 1.51-1.53 (2 H, m), 1.28 (3 H, t, J = 7.11 Hz).

MS (ESI): *m/z* 334.13271 M + H (calculated 334.13168)

HPLC (method A) t_R = 10.55 min (99% pure).

7-Chloro-*N*-(piperidin-4-yl)quinolin-4-amine (Iwasaki, 1994)

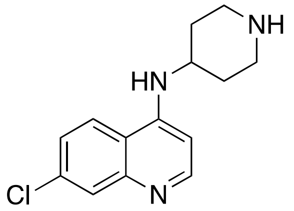

Ethyl 4-((7-chloroquinolin-4-yl)amino)piperidine-1-carboxylate (2.45 g, 7.3 mmol, the product of multiple reactions), 95% ethanol (100 mL), and 10% caustic soda (4.5 mL) were allowed to heat, stirring, at reflux for 4 days. As TLC indicated that the reaction was not complete, 50% caustic soda (0.5 mL) was added, and reflux was continued for a further 3 days. TLC then indicated that the reaction was complete. The reaction solvent was removed under reduced pressure with warming, and the residue was partitioned between chloroform (20 mL) and water (50 mL). After separation, the aqueous layer was extracted with additional chloroform (3 x 10 mL), and the pooled organic layers were dried ($MgSO_4$) and concentrated under reduced pressure with warming to yield a tan solid (1.05 g). A cream colored solid was also isolated from the aqueous layer by vacuum filtration (1.04 g). NMR indicated that both solids obtained were the desired product (total yield 1.83 g, 96%, mp = 166.3-169.4°C).

^{1}H NMR δ (ppm)($CDCl_3$): 8.52 (1 H, d, J = 5.39 Hz), 7.96 (1 H, d, J = 2.18 Hz), 7.65 (1 H, d, J = 8.96 Hz), 7.37 (1 H, dd, J = 8.93, 2.19 Hz), 6.45 (1 H, d, J = 5.42 Hz), 4.87 (1 H, br d, J = 7.35 Hz), 3.61-3.62 (1 H, m), 3.19 (2 H, dt, J = 12.68, 3.70 Hz), 2.79-2.81 (2 H, m), 2.15-2.19 (2 H, m), 1.50-1.50 (2 H, m).

MS (ESI): *m/z* 262.11116 M + H (calculated 262.11065)

HPLC (method A) t_R = 2.74 min (94% pure).

3-Carbethoxy-4-quinolone (Claisen, 1897; Gould, 1939; Price, 1946; Hauser, 1950; De, 1998)

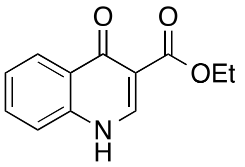

Aniline (10.0 mL, 110 mmol) and diethyl ethoxymethylene malonate (21.99 mL, 110 mmol) were heated in refluxing Dowtherm A (100 mL) for 30 minutes. After cooling, the reaction mixture was diluted with hexanes (150 mL) and vacuum filtered, rinsing with further hexanes (200 mL), followed by acetone (100 mL). The product was obtained as grayish beige, powdery solid (13.87 g, 58%).

^{1}H NMR δ (ppm)(DMSO-d_6): 12.31 (1 H, s), 8.54 (1 H, s), 8.16 (1 H, dd, J = 8.09, 1.47 Hz), 7.71 (1 H, ddd, J = 8.27, 6.99, 1.54 Hz), 7.62 (1 H, dd, J = 8.24, 1.07 Hz), 7.42 (1 H, ddd, J = 8.08, 6.99, 1.17 Hz), 4.22 (2 H, q, J = 7.10 Hz), 1.28 (3 H, t, J = 7.10 Hz).

3-Carboxy-8-chloro-4-quinolone (Price, 1946; Hauser, 1950; De, 1998)

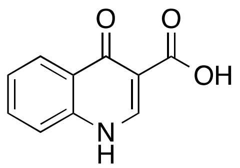

3-Carbethoxy-4-quinolone (13.87 g, 63.9 mmol) was heated at reflux in 200 mL of 10% caustic soda for 18 hours. The hot reaction mixture was poured into 300 mL water, then made acidic by the addition of concentrated muriatic acid. After cooling, vacuum filtration followed by air-drying provided the desired product as a white solid (12.20 g, containing residual water). This material was used without further drying in the ensuing reaction.

^{1}H NMR δ (ppm)(DMSO-d_6): 15.36 (1 H, br s), 13.48 (1 H, br s), 8.90 (1 H, s), 8.31 (1 H, d, J = 8.15 Hz), 7.90 (1 H, m), 7.84 (1 H, d, J = 8.35 Hz), 7.62 (1 H, m).

4-Quinolone (Price, 1946; Hauser, 1950; De, 1998)

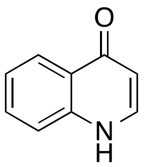

3-Carboxy-4-quinolone (12.06 g, containing residual water) was heated in Dowtherm A (100 mL) at reflux for 40 minutes. After cooling, the reaction mixture was diluted with 100 mL hexanes and allowed to sit for 48 hours, followed by vacuum filtration, rinsing with further hexanes (300 mL) to provide the product as an off-white, powdery solid (9.28 g, >99% from 3-carbethoxy-4-quinolone).

^{1}H NMR δ (ppm)(DMSO-d_6): 11.74 (1 H, s), 8.09 (1 H, dd, J = 8.08, 1.47 Hz), 7.89 (1 H, dd, J = 7.39, 5.89 Hz), 7.64 (1 H, ddd, J = 8.34, 6.94, 1.56 Hz), 7.54 (1 H, d, J = 8.31 Hz), 7.31 (1 H, ddd, J = 8.09, 6.94, 1.12 Hz), 6.03 (1 H, dd, J = 7.38, 1.22 Hz).

4-Chloroquinoline (Price, 1946; Hauser, 1950; De, 1998)

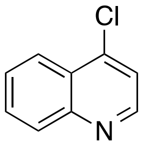

4-Quinolone (9.28 g, 63.9 mmol) and phosphorus oxychloride (35 mL, 375 mmol) were heated at 110ºC for 2.5 hours, whereupon TLC indicated that the reaction was complete. After cooling, the reaction mixture was poured cautiously onto ice, stirring vigorously. After resting, the aqueous mixture was made basic to litmus by the addition of solid caustic potash (final volume 800 mL). After cooling, this mixture was extracted with chloroform (200 mL followed by 3 x 20 mL), and the pooled organic layers were dried ($MgSO_4$) and evaporated under reduced pressure with warming to yield the desired product as a viscous, aromatic liquid. Upon storage for several years in a sealed vial, this crystallized to a beige solid (9.42 g, 90%, mp = 25.9-28.1°C (lit. 34-35°C; Hauser, 1950)).

^{1}H NMR δ (ppm)($CDCl_3$): 8.80 (1 H, d, J = 4.70 Hz), 8.25 (1 H, dd, J = 8.42, 1.38 Hz), 8.14 (1 H, dd, J = 8.48, 1.11 Hz), 7.79 (1 H, ddd, J = 8.49, 6.89, 1.44 Hz), 7.66 (1 H, ddd, J = 8.42, 6.90, 1.23 Hz), 7.51 (1 H, d, J = 4.70 Hz).

3-(Quinolin-4-ylamino)propanol (Burgess, 2006; Burgess, 2010)

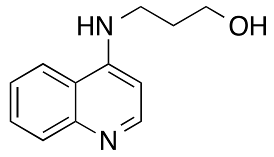

4-Chloroquinoline (3.00 g, 18.3 mmol) and 3-amino-1-propanol (8.42 mL, 110 mmol) were heated at 70°C in a Carius vessel for 24 hours. TLC indicated that the reaction was not complete. The temperature was increased to 90°C, and heating was continued for a further 6 days. Reaction was found to be complete by TLC, and the thick reaction mixture was poured without cooling into water (300 mL). After resting for 20 minutes, the resulting precipitate was recovered by vacuum filtration to give the desired product as a creamy white, sparkling solid (3.60 g, 97%, mp = 148.7-149.7°C).

^{1}H NMR δ (ppm)(DMSO-d_6): 8.38 (1 H, d, J = 5.30 Hz), 8.18 (1 H, dd, J = 8.42, 1.33 Hz), 7.76 (1 H, dd, J = 8.37, 1.26 Hz), 7.59 (1 H, ddd, J = 8.39, 6.77, 1.37 Hz), 7.40 (1 H, ddd, J = 8.40, 6.77, 1.35 Hz), 7.13 (1 H, t, J = 5.39 Hz), 6.44 (1 H, d, J = 5.35 Hz), 4.59 (1 H, t, J = 5.06 Hz), 3.55 (2 H, m), 3.32-3.36 (water signal overlaps m, ~2 H), 1.83 (2 H, m).

MS (ESI): *m/z* 203.11814 M + H (calculated 203.11789)

HPLC (method A) t_R = 6.47 min (99% pure).

3-(Quinolin-4-ylamino)propyl methanesulfonate (Burgess, 2006; Burgess, 2010; Andrews, 2010)

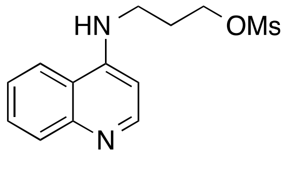

3-(quinolin-4-ylamino)propanol (0.50 g, 2.5 mol) and triethylamine (0.52 mL, 3.7 mmol) were taken up in 50 mL anhydrous dichloromethane and chilled to below 0°C in an ice-salt bath. Methanesulfonyl chloride (0.22 mL, 2.8 mmol) was gradually added and stirring on ice was continued for 1 hour. TLC indicated that the reaction was not complete. The flask was sealed and stored at 5°C for 18 hours. Further triethylamine (1.00 mL, 7.2 mmol) and methanesulfonyl chloride (0.12 mL, 1.5 mmol) were then added. After thirty minutes, TLC indicated that reaction was complete. The reaction mixture was washed with saturated $NaHCO_3$ (25 mL), and the aqueous layer was then extracted with dichloromethane (3 x 7 mL). The pooled organic layers were dried over magnesium sulfate and evaporated under reduced pressure with warming to yield a cream colored solid (0.61 g, 88%).

^{1}H NMR δ (ppm)(DMSO-d_6): 8.40 (1 H, d, J = 5.31 Hz), 8.20 (1 H, dd, J = 8.45, 1.31 Hz), 7.78 (1 H, dd, J = 8.39, 1.25 Hz), 7.61 (1 H, ddd, J = 8.42, 6.78, 1.31 Hz), 7.42 (1 H, ddd, J = 8.42, 6.78, 1.33 Hz), 7.17 (1 H, br t, J = 5.45 Hz), 6.48 (1 H, d, J = 5.36 Hz), 4.35 (2 H, t, J = 6.21 Hz), 3.39 (2 H, m), 3.20 (3 H, s), 2.08 (2 H, m).

Ethyl 4-(quinolin-4-ylamino)piperidine-1-carboxylate (Andersag, 1948; Surrey, 1951)

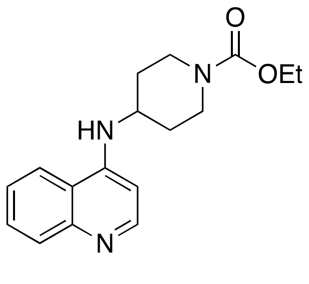

4-Chloroquinoline (2.00 g, 12 mmol), ethyl 4-amino-1-piperidine carboxylate (3.16 g, 32 mmol), and phenol (6.90 g, 73 mmol) were heated at 90ºC in a sealed Carius vessel for 6 days. TLC indicated that unreacted 4,7-dichloroquinoline remained, and therefore additional ethyl 4-amino-1-piperidine carboxylate (1.00 g, 1.0 mmol) was added. The vessel was again sealed and heated for a further 2 days, whereupon TLC indicated that reaction was complete. The reaction mixture was diluted with chloroform (50 mL) and rinsed with 10% caustic soda (5 x 10 mL), followed by further rinsing with water (3 x 10 mL). The organic layer was dried over $MgSO_4$ and concentrated under reduced pressure with warming to yield a brown semisolid. This material was taken up in boiling ethyl acetate (70 mL) and allowed to cool and concentrate at room temperature (to approximately 15 mL). Vacuum filtration provided the desired product as a sparkling, tan, crystalline solid (2.81 g, 77%, mp = 209-210°C).

^1H NMR δ (ppm)($CDCl_3$): 8.56 (1 H, d, J = 5.31 Hz), 7.99 (1 H, dd, J = 8.44, 1.20 Hz), 7.72 (1 H, dd, J = 8.43, 1.25 Hz), 7.64 (1 H, ddd, J = 8.46, 6.85, 1.31 Hz), 7.44 (1 H, ddd, J = 8.40, 6.86, 1.29 Hz), 6.47 (1 H, d, J = 5.35 Hz), 4.90 (1 H, br d, J = 7.27 Hz), 4.17 (4 H, br s overlaps q, J = 7.11 Hz), 3.70-3.71 (1 H, m), 3.06 (2 H, td, J = 12.54, 2.73 Hz), 2.13-2.22 (2 H, m), 1.52-1.54 (2 H, m), 1.28 (3 H, t, J = 7.11 Hz).

MS (ESI): *m/z* 300.17103 M + H (calculated 300.17065)

HPLC (method A) t_R = 9.81 min (99% pure).

***N*-(Piperidin-4-yl)quinolin-4-amine** (Iwasaki, 1994)

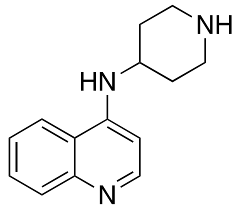

Ethyl 4-(quinolin-4-ylamino)piperidine-1-carboxylate (1.77 g, 5.9 mmol), 95% ethanol (40 mL), and 50% caustic soda (2.00 mL) were allowed to heat, stirring, at reflux for 20 hours. As TLC indicated that the reaction was not complete, additional 50% caustic soda (1.0 mL) was added, and reflux was continued for a further 3 days. TLC then indicated that the reaction was complete. The reaction solvent was removed under reduced pressure with warming, and the residue was partitioned between chloroform

(25 mL) and water (25 mL). After separation, the aqueous layer was extracted with additional chloroform (3 x 7 mL). The pooled organic layers were vacuum filtered to remove solid material, followed by drying ($MgSO_4$) and concentration under reduced pressure with warming to yield the desired product as a white powder (0.65 g, 48%, mp = 112.5-124°C).

^{1}H NMR δ (ppm)($CDCl_3$): 8.55 (1 H, d, J = 5.33 Hz), 7.98 (1 H, dd, J = 8.45, 1.22 Hz), 7.72 (1 H, dd, J = 8.42, 1.26 Hz), 7.63 (1 H, ddd, J = 8.45, 6.85, 1.33 Hz), 7.43 (1 H, ddd, J = 8.39, 6.85, 1.30 Hz), 6.47 (1 H, d, J = 5.36 Hz), 4.89 (1 H, br d, J = 7.32 Hz), 3.63-3.64 (1 H, m), 3.19 (2 H, dt, J = 12.67, 3.73 Hz), 2.80-2.81 (2 H, m), 2.17-2.20 (2 H, m), 1.44-1.56 (2 H, m).

MS (ESI): *m/z* 228.14987 M + H (calculated 228.14952)

HPLC (method A) t_R = 1.58 min (78% pure). (Second largest peak elutes at 2.00 minutes, 17%.)

3-Carbethoxy-8-trifluoromethyl-4-quinolone (Claisen, 1897; Gould, 1939; Price, 1946; Allais, 1969; De, 1998)

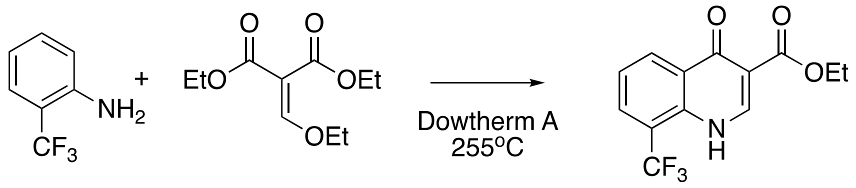

ortho-Trifluoromethyl aniline (7.09 mL, 56.4 mmol), diethyl ethoxymethylene malonate (11.63 mL, 58.4 mmol, 1.04 eq), and a catalytic amount of *para*-toluenesulfonic acid were heated at reflux in benzene (80 mL) for 24 hours. The solvent was removed under reduced pressure with warming to afford a golden brown, crystalline solid. This was dissolved in 10 mL Dowtherm A at 150°C, and then added dropwise to 80 mL of boiling Dowtherm A (255°C) in an open 3-necked flask, taking care that temperature did not drop below 250°C during addition (10 minutes). The reaction was allowed to stir at 255°C for a further 15 minutes, and then removed from the heat. After cooling, the reaction mixture was diluted with hexanes (125 mL) and vacuum filtered, rinsing with additional hexanes (300 mL) followed by acetone (10 mL) to obtain the desired product as an off-white, crystalline solid (14.15 g; 86%).

^{1}H NMR δ (ppm)(DMSO-d_6): 11.68 (1H, br s), 8.54-8.40 (2H, m), 8.14 (1H, d, J = 7.45 Hz), 7.65-7.54 (1H, m), 4.25 (2H, q, J = 7.05 Hz), 1.30 (3H, t, 7.07 Hz).

^{19}F NMR δ (ppm)(DMSO-d_6): -58.89.

3-Carboxy-8-trifluoromethyl-4-quinolone (Price, 1946; Allais, 1969; De, 1998)

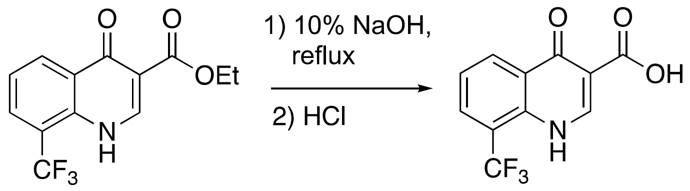

3-Carbethoxy-8-trifluoromethyl-4-quinolone (14.10 g, 49.4 mmol) was allowed to reflux in 10% caustic soda (150 mL) for 1.5 hours. The hot reaction mixture was poured into 400 mL water and made acidic by addition of concentrated muriatic acid. The resulting mixture was allowed to stand for 18 hours, followed by filtration, rinsing with 400 mL water. After air-drying, the desired product was obtained as a fine, white solid (12.19 g; 97%).

^{1}H NMR δ (ppm)(DMSO-d_6): 14.66 (1H, br s), 12.62 (1H, br s), 8.66 (1H, s), 8.61 (1H, d, J = 8.20 Hz), 8.32 (1H, d, J = 7.39 Hz), 7.79-7.71 (1H, m).

^{1}H NMR δ (ppm)(DMSO-d_6): -58.78.

8-Trifluoromethyl-4-quinolone (Price, 1946; Allais, 1969; De, 1998)

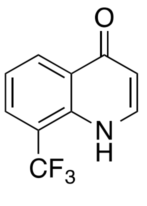

3-Carboxy-8-trifluoromethyl-4-quinolone (12.00 g, 46.7 mmol) was heated in Dowtherm A (100 mL) at 255°C for 25 minutes, stirring, in an open, 3-necked flask. After cooling, the reaction mixture was diluted with hexanes (100 mL) and vacuum filtered, rinsing

with additional hexanes (250 mL) to afford the desired product as a beige, crystalline solid (9.53 g, 95%).

^1H NMR δ (ppm)(DMSO-d_6): 11.22 (1 H, br s), 8.44 (1H, d, J = 8.05 Hz), 8.09 (1H, d, J = 7.24 Hz), 7.96-7.81 (1H, m), 7.56-7.43 (1H, m), 6.20 (1H, d, J = 6.92 Hz).

^{19}F NMR δ (ppm)(DMSO-d_6): -59.38.

4-Chloro-8-(trifluoromethyl)quinoline (Price, 1946; Allais, 1969; De, 1998)

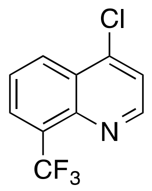

Phosphorus oxychloride (35 mL, 380 mmol, 8.5 eq) was cautiously added to 8-trifluoromethyl-4-quinolone (9.40 g, 44.1 mmol), followed by heating at 110°C for 2 hours. After cooling, the reaction mixture was gradually and cautiously poured onto ice with vigorous stirring (note: delayed exothermic reaction). After resting, the inhomogeneous mixture was made basic by the addition of 50% caustic soda, adding additional ice to control the resulting exothermic reaction. After standing 18 hours, the mixture (1000 mL) was extracted with chloroform (100 mL, followed by 3 x 50 mL). The pooled organic layers were dried ($MgSO_4$) and concentrated under reduced pressure with warming to afford the desired product as a beige, crystalline solid with a characteristic odor of 4-chloroquinoline (9.37 g, 92%).

^1H NMR δ (ppm)($CDCl_3$): 8.94 (1 H, d, J = 4.71 Hz), 8.48 (1 H, d, J = 8.55 Hz), 8.15 (1 H, d, J = 7.25 Hz), 7.74-7.67 (1 H, m), 7.61 (1 H, d, J = 4.72 Hz).

^{19}F NMR δ (ppm)($CDCl_3$): -59.65.

3-(8-(Trifluoromethyl)quinolin-4-ylamino)propanol (Burgess, 2006; Burgess, 2010)

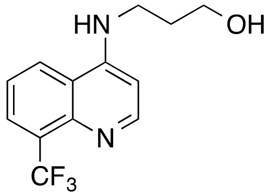

4-Chloro-8-(trifluoromethyl)quinoline (20.49 g, 88.5 mmol) and 3-amino-1-propanol (43.0 mL, 562 mmol) were allowed to heat at 110°C for 1.5 hours, at the end of which time TLC indicated that reaction was complete. The hot reaction mixture was poured into 500 mL deionized water, agitating vigorously with a stirring rod, and the resulting precipitate was recovered by vacuum filtration. Upon drying, the desired product was obtained as an off-white powder (21.53 g, 90%, mp = 162.6-163.7°C).

^{1}H NMR δ (ppm)(DMSO-d_6): 8.52 (1 H, d, J = 8.53 Hz), 8.49 (1 H, d, J = 5.43 Hz), 8.01 (1 H, d, J = 7.29 Hz), 7.52 (1 H, t, J = 7.88 Hz), 7.42 (1 H, t, J = 5.37 Hz), 6.58 (1 H, d, J = 5.49 Hz), 4.60 (1 H, t, J = 5.06 Hz), 3.55 (2 H, m), 3.36-3.38 (water signal overlaps m, ~2 H), 1.79-1.87 (2 H, m).

^{1}H NMR δ (ppm)($CDCl_3$): 8.69 (1 H, d, J = 5.37 Hz), 7.96 (2 H, m), 7.43 (1 H, t, J = 7.89 Hz), 6.50 (1 H, d, J = 5.40 Hz), 6.05 (1 H, br s), 3.98 (2 H, t, J = 5.39 Hz), 3.49 (2 H, m), 2.06 (2 H, m).

^{19}F NMR δ (ppm)(DMSO-d_6): -58.6.

MS (ESI): *m/z* 271.10583 M + H (calculated 271.10527)

HPLC (method A) t_R = 7.98 min (>99% pure).

3-(8-Trifluoromethyl-quinolin-4-ylamino)propyl methanesulfonate (Burgess, 2006; Burgess, 2010; Andrews, 2010)

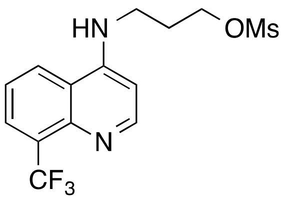

3-(8-(Trifluoromethyl)quinolin-4-ylamino)propanol (21.53 g, 79.7 mmol) and triethylamine (20.0 mL, 140 mmol) in anhydrous chloroform (500 mL) were cooled to below 0°C on ice/salt, and methanesulfonyl chloride (12.0 mL, 154 mmol) was gradually added. After stirring 1.5 hours on ice, TLC indicated that reaction was not complete. Additional methanesulfonyl chloride (1.70 mL, 2.19 mmol) was added; after an additional hour of stirring, TLC indicated that reaction was complete. The reaction mixture was shaken with saturated sodium bicarbonate (150 mL), and the aqueous layer was then extracted with 3 x 20 mL chloroform. Upon standing, a fine, needlelike precipitate appeared in the pooled organic layers; this was recovered by filtration. NMR indicated that this material was the desired product (a white, crystalline solid, 5.87 g, mp = 141.6-142.8°C). The filtrate was dried over magnesium sulfate and evaporated under reduced pressure with warming to yield a tan solid. NMR indicated that this, too, was the desired product (20.97 g; total yield 26.84 g, 97%).

^{1}H NMR δ (ppm)(DMSO-d_6): 8.50-8.51 (2 H, m), 8.03 (1 H, d, J = 7.28 Hz), 7.54 (1 H, t, J = 7.88 Hz), 7.46 (1 H, t, J = 5.41 Hz), 6.62 (1 H, d, J = 5.47 Hz), 4.36 (2 H, t, J = 6.18 Hz), 3.42 (2 H, m), 3.20 (3 H, s), 2.05-2.13 (2 H, m).

^{19}F NMR δ (ppm)(DMSO-d_6): -58.6.

MS (ESI): *m/z* 349.08340 M + H (calculated 349.08282)

HPLC (method A) t_R = 9.28 min (99% pure)

Note: HPLC and MS data are of the precipitate, which is presumed to be of atypically high purity for a 3-(quinoline-4-ylamino)propyl methanesulfonate produced by this method.

Ethyl 4-((8-(trifluoromethyl)quinolin-4-yl)amino)piperidine-1-carboxylate (Andersag, 1948; Surrey, 1951)

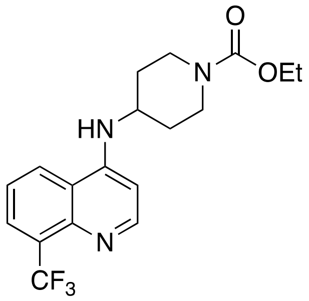

4-Chloro-(8-trifluoromethyl)quinoline (1.50 g, 6.5 mmol), ethyl 4-amino-1-piperidine carboxylate (1.02 g, 5.9 mmol), and phenol (3.04 g, 32 mol) were heated at 82ºC in a sealed Carius vessel for 20 hours. Additional ethyl 4-amino-1-piperidine carboxylate (0.20 g, 1.2 mmol) was added. The vessel was again sealed and heated for a further 3 days at 100ºC. whereupon TLC indicated that no unreacted quinoline remained. The reaction mixture was diluted with chloroform (25 mL) and rinsed with 10% 8 soda (5 x 10 mL), followed by further rinsing with brine (2 x 10 mL). The organic layer was dried over $MgSO_4$ and concentrated under reduced pressure with warming to yield a thick, brown oil. This was diluted with dichloromethane and adsorbed onto silica, then purified by flash chromatography on silica, eluting with a gradient of 100% hexanes to 100% ethyl acetate. The desired product was obtained as a beige solid (0.63 g, 27%, R_f = 0.4 (silica, ethyl acetate)). The quinoline phenolate was also isolated from chromatography (a sparkling, white, crystalline solid, 0.94 g, 50%, mp = 175.4-181.0ºC, Rf = 0.7 (silica, ethyl acetate)).

^{19}F NMR δ (ppm)($CDCl_3$): -60.8.

MS (ESI): *m/z* 368.15863 M + H (calculated 368.15804)

HPLC (method A) t_R = 10.69 min (96% pure).

***N*-(Piperidin-4-yl)-8-(trifluoromethyl)quinolin-4-amine** (Iwasaki, 1994)

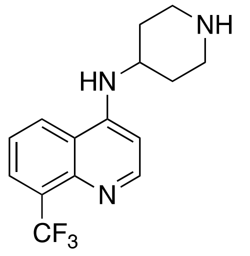

Ethyl 4-((8-(trifluoromethyl)quinolin-4-yl)amino)piperidine-1-carboxylate (0.62 g, 1.7 mmol), 95% ethanol (50 mL), and 10% caustic soda (4 mL) were allowed to heat, stirring, at reflux for 18 hours. As TLC indicated that the reaction was not complete, 50% caustic soda (0.5 mL) was added, and reflux was continued for a further 24 hours. TLC then indicated that the reaction was complete. After removal of the solvent under reduced pressure with warming, the residue was partitioned between chloroform (25 mL) and water (50 mL). After separation, the aqueous layer was extracted with additional chloroform (3 x 10 mL), and the pooled organic layers were dried ($MgSO_4$) and concentrated under reduced pressure with warming to provide a pale yellow solid (0.18 g, 36%, mp = 186.8-189.6°C).

^{1}H NMR δ (ppm)($CDCl_3$): 8.69 (1 H, d, J = 5.40 Hz), 8.00 (1 H, d, J = 7.33 Hz), 7.93 (1 H, d, J = 8.49 Hz), 7.46 (1 H, dd, J = 8.45, 7.34 Hz), 6.56 (1 H, d, J = 5.43 Hz), 4.93 (1 H, br d, J = 7.30 Hz), 3.63-3.64 (1 H, m), 3.20 (2 H, m), 2.79-2.80 (2 H, m), 2.16-2.20 (2 H, m), 1.49-1.50 (2 H, m).

MS (ESI): *m/z* 296.13763 M + H (calculated 296.13691)

HPLC (method A) t_R = 2.85 min (92% pure). (Additional peaks elute at 1.81 minutes, 2%, and 7.54 minutes, 1%.)

Compound 1: 1,3-bis(4-(quinolin-4-yl)piperazin-1-yl)propane (Abel, 1996; Ghosh, 2010)

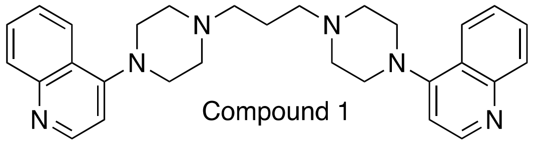

Compound 1

1,3-Dibromopropane (0.92 g, 0.0045 mol) and $NaHCO_3$ (0.92 g, 0.011 mol) were added to water (30 mL), and 4-(piperazin-1-yl)quinoline (Abel, 1996; Ghosh, 2010) (1.9 g, 0.009 mol) was added. The reaction was allowed to reflux for 15 hours. Upon cooling, the solid was recovered by filtration, and washed with water until the pH of the filtrate was neutral. This solid was heated in ethanol (20 mL) at 60°C for 2 hours, allowed to cool, and filtered. The resulting solid was dried in a vacuum desiccator to afford the desired product (0.52 g, 25%).

HPLC (method A) t_R = 5.61 min (91% pure).

^{1}H NMR δ (ppm)($CDCl_3$): 8.71-8.76 (2 H, m), 7.98-8.09 (4 H, m), 7.66 (2 H, ddd, J = 8.46, 6.80, 1.45 Hz), 7.46-7.52 (2 H, m), 6.86 (2 H, t, J = 5.01 Hz), 3.29 (8 H, br s), 2.78 (8 H, br s), 2.57 (4 H, t, J = 7.49 Hz), 1.79-1.90 (2 H, m).

^{13}C NMR δ (ppm)($CDCl_3$): 156.9, 150.9, 149.6, 130.0, 129.0, 123.7, 123.5, 125.3, 108.7, 56.7, 53.3, 52.2, 24.4.

MS (ESI): *m/z* 467.2916 M + H (Calculated 467.2918).

Compound 5: *N,N'*-(piperazine-1,4-diylbis(propane-3,1-diyl))bis(7-chloroquinolin-4-amine) (Andersag, 1948; Surrey, 1951; this compound originally reported: Rhône-Poulenc, 1962.)

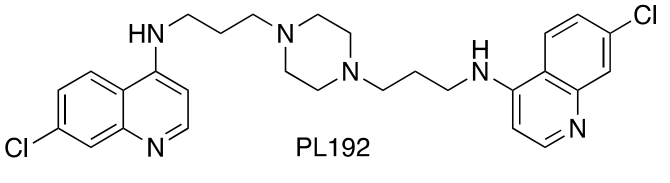

PL192

1,4-Bis(3-aminopropyl)piperazine (0.79 g, 3.9 mmol), 4,7-dichloroquinoline (1.64 g, 8.3 mmol, 2.1 eq), and phenol (6.0 g, 64 mmol, 16 eq) were heated at 125°C for 4 hours. Upon cooling, the reaction mixture was diluted with dichloromethane (40 mL) and

washed with 2M caustic soda (6 x 20 mL), followed by brine (30 mL). After drying over $MgSO_4$ and evaporation under reduced pressure with warming, the crude product was recrystallized from 25:75 methanol:ethyl acetate. The desired compound was obtained as an off-white, crystalline solid (0.02 g, 1%).

^{1}H NMR δ (ppm)($CDCl_3$): 8.53 (2 H, d, J = 5.35 Hz), 7.95 (2 H, dd, J = 6.47, 2.15 Hz), 7.91-7.83 (2 H, m), 7.37 (2 H, s), 7.37-7.28 (2 H, m), 6.40-6.32 (2 H, m), 3.47-3.36 (4 H, m), 2.78-2.72 (8 H, m), 2.06-1.96 (4 H, m), 1.71 (4 H, m).

^{13}C NMR δ (ppm)($CDCl_3$): 152.3, 150.5, 149.2, 134.6, 128.8, 124.7, 122.2, 117.5, 98.7, 59.0, 53.7, 44.3, 23.6.

HPLC (method B) t_R = 6.38 (96% pure).

MS (ESI): *m/z* 523.2147 M + H (calculated 523.2138).

Compound 7: *N,N'*-(3,3'-(piperazine-1,4-diyl)bis(propane-3,1-diyl))diquinolin-4-amine (Andersag, 1948; Surrey, 1951)

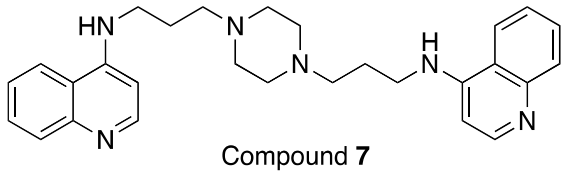

Compound **7**

1,4-Bis(3-aminopropyl)piperazine (0.87 g, 4.4 mmol), 4-chloroquinoline (1.5 g, 9.2 mmol, 2.1 eq), and 6 g phenol (64 mmol, 15 eq) were heated at 125°C for 4 hours. Upon cooling, the reaction mixture was diluted with dichloromethane (40 mL) and washed with 2M caustic soda (6 x 20 mL), followed by brine (30 mL). Following drying ($MgSO_4$) and evaporation under reduced pressure with warming, the crude product was recrystallized from ethyl acetate. The desired product was obtained as a white, crystalline solid (0.13 g, 7%).

^{1}H NMR δ (ppm)($CDCl_3$): 8.55 (2 H, d, J = 5.31 Hz), 7.98 (2 H, dd, J = 8.47, 1.18 Hz), 7.94 (2 H, d, J = 8.38 Hz), 7.63 (2 H, ddd, J = 8.45, 6.83, 1.33 Hz), 7.41 (2 H, ddd, J = 8.38, 6.82, 1.28 Hz), 7.25 (2 H, s), 6.38 (2 H, d, J = 5.36 Hz), 3.44 (4 H, m, J = 5.29 Hz), 2.77-2.71 (12H, m), 2.07-1.99 (4 H, m).

HPLC (method B) t_R =4.73 (95% pure).

MS (ESI): *m/z* 382.1784 M + H (calculated 382.1793).

Compound 12: 7-chloro-*N*-(1-(3-(quinolin-4-ylamino)propyl)piperidin-4-yl)quinolin-4-amine (Burgess, 2010)

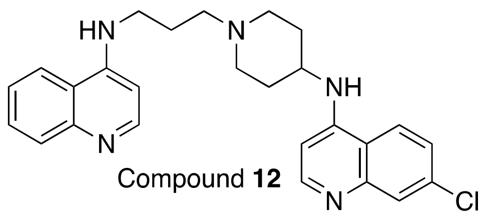

3-(Quinolin-4-ylamino)propyl methanesulfonate (0.61 g, 2.2 mol), 7-chloro-*N*-(piperidin-4-yl)quinolin-4-amine (0.60 g, 2.3 mol), K_2CO_3 (0.45 g, 3.3 mol), and a catalytic amount of potassium iodide were heated for 5 days in refluxing acetonitrile (25 mL), at which point TLC indicated that reaction was complete. The solvent was removed under reduced pressure with warming (after dilution with 10 mL water), and the residue was combined with water (50 mL) and chloroform (80 mL). A large amount of material remained undissolved and was recovered by vacuum filtration (a cream colored solid). The filtrate was placed in a separatory funnel and separated, and the aqueous layer was extracted thrice with 10 mL portions of chloroform. The pooled chloroform layers were dried ($MgSO_4$) and evaporated under reduced pressure with warming to yield a cloudy, pale yellow oil, 0.07 g. NMR indicated that the oil from extraction contained both product and starting material, whereas the solid recovered from filtration was the desired product with very little impurity. The latter was taken up in excess boiling 95% ethanol, and allowed to evaporate gradually (at room temperature) to ~10 mL. Filtration afforded the desired product as beige crystals (0.79 g, 81%, mp = 240-250°C (dec)).

^{1}H NMR δ (ppm)(DMSO-d_6): 8.37-8.41 (3 H, m), 8.19 (1 H, d, J = 8.41 Hz), 7.77-7.79 (2 H, m), 7.61 (1 H, ddd, J = 8.43, 6.73, 1.31 Hz), 7.42-7.46 (2 H, m), 7.30 (1 H, br t, J = 5.24 Hz), 6.99 (1 H, br d, J = 7.49 Hz), 6.55 (1 H, d, J = 5.54 Hz), 6.48 (1 H, d, J = 5.37 Hz), 3.50-3.53 (1 H, m), 3.41-3.46 (2 H, m), 2.95-2.98 (2 H, m), 2.46 (2 H, t, J = 6.79 Hz), 2.06-2.12 (2 H, m), 1.98-2.01 (2 H, m), 1.85 (2 H, m), 1.63-1.72 (2 H, m).

^{13}C NMR δ (ppm)(DMSO-d_6): 151.8, 150.6, 149.9, 149.2, 149.1, 148.2, 133.3, 128.9, 128.6, 127.4, 124.4, 123.8, 123.7, 121.5, 118.8, 117.4, 99.1, 98.0, 55.7, 52.3, 49.6, 40.9, 30.9, 25.3.

MS (ESI): *m/z* 446.21096 M + H (calculated 446.21060)

HPLC (method A) t_R = 6.07 min (99% pure).

Compound 11: 7-chloro-*N*-(3-(4-(quinolin-4-ylamino)piperidin-1-yl)propyl)quinolin-4-amine (Burgess, 2010)

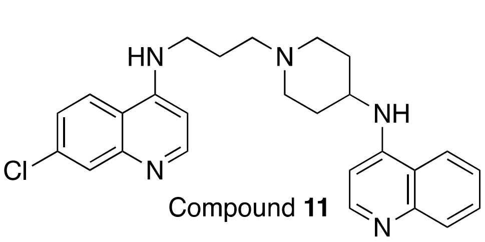

Compound **11**

3-(7-Chloroquinolin-4-ylamino)propyl methanesulfonate (0.83 g, 0.00264 mol), *N*-(piperidin-4-yl)quinolin-4-amine (0.63 g, 0.00277 mol), K_2CO_3 (0.55 g, 0.00396 mol), and a catalytic amount of potassium iodide were heated for 24 hours in refluxing acetonitrile (50 mL), at which point TLC indicated that reaction was complete. After dilution with 10 mL water, the solvent was removed under reduced pressure with warming. The residue was combined with additional water (30 mL in total) and chloroform (30mL). A cream colored solid remained undissolved, and was recovered by vacuum filtration. The filtrate was separated into two layers, and the aqueous layer was extracted with chloroform (3 x 10 mL). The pooled organic layers were dried ($MgSO_4$) and evaporated under reduced pressure with warming to yield a waxy, yellow solid. This solid, together with the solid obtained from filtration, was dissolved in excess boiling 95% ethanol (100 mL) and allowed to evaporate gradually (at room temperature) to ~ 10 mL. Filtration afforded the desired product as a beige solid (0.66 g, 56%, mp = 224-228°C).

^1H NMR δ (ppm)($CDCl_3$): 8.58 (1 H, d, J = 5.28 Hz), 8.54 (1 H, d, J = 5.34 Hz), 8.01 (1 H, dd, J = 8.43, 1.20 Hz), 7.97 (1 H, d, J = 2.14 Hz), 7.77-7.81 (2 H, m), 7.68 (1 H, ddd, J = 8.46, 6.84, 1.29 Hz), 7.51 (1 H, ddd, J = 8.38, 6.84, 1.30 Hz), 7.37 (1 H, dd, J = 8.88, 2.16 Hz), 7.11 (1 H, br t, J = 4.14 Hz), 6.49 (1 H, d, J = 5.35 Hz), 6.38 (1 H, d, J = 5.38 Hz), 4.93 (1 H, br d, J = 6.80 Hz), 3.66-3.69 (1 H, m), 3.43 (2 H, td, J = 5.97, 4.29 Hz), 3.05-3.08 (2 H, m), 2.68 (2 H, t, J = 5.61 Hz), 2.30 (4 H, d, J = 14.10 Hz), 1.99 (2 H, m), 1.72-1.81 (2 H, m).

^{13}C NMR δ (ppm)(DMSO-d_6): 151.9, 150.7, 150.1, 149.1, 149.0, 148.5, 133.3, 129.0, 128.7, 127.5, 124.1, 124.0, 123.6, 122.0, 118.9, 117.4, 98.6, 98.6, 55.7, 52.4, 49.5, 41.0, 31.1, 25.3.

MS (ESI): *m/z* 446.21091 M + H (calculated 446.21060)

HPLC (method A) t_R = 6.35 min (93% pure). (An additional peak elutes at 6.94 minutes, 6%.)

Compound 13: *N*-(3-(4-(quinolin-4-ylamino)piperidin-1-yl)propyl)quinolin-4-amine (Burgess, 2010)

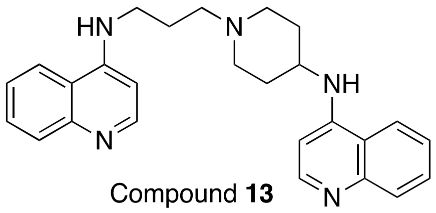

Compound **13**

3-(Quinolin-4-ylamino)propyl methanesulfonate (0.76 g, 0.00271 mol), *N*-(piperidin-4-yl)quinolin-4-amine (0.65 g, 0.00285 mol), K_2CO_3 (0.56 g, 0.00407 mol), and a catalytic amount of potassium iodide were heated for 24 hours in refluxing acetonitrile (40 mL), at which point TLC indicated that reaction was complete. After dilution with 10 mL water, the acetonitrile was removed by evaporation under reduced pressure with warming, and the residue was combined with dichloromethane (30 mL) and water (30 mL). An undissolved tan solid was removed by filtration, rinsing with dichloromethane and water. The dichloromethane and water layers were separated, and the aqueous layer was extracted with dichloromethane (3 x 10 mL), followed by drying of the pooled organic layers ($MgSO_4$) and evaporation under reduced pressure with warming to yield a yellow solid. NMR indicated that this was primarily starting material, whereas the solid from filtration was primarily the desired product. The latter was dissolved in excess boiling 95% ethanol, and allowed to evaporate gradually (at room temperature) to approximately 10 mL. Filtration afforded a tan solid (0.80 g). This material was further purified by automated flash chromatography on alumina, eluting with a gradient of 100% hexanes to 100% ethyl acetate. The desired product was

obtained as a white solid (0.29 g, 26%, mp =190.2-191.8°C, R_f =0.14 (alumina, 10/90 MeOH/EA v/v)).

1H-NMR (600 MHz; DMSO-d6): δ 8.40 (d, J = 5.3 Hz, 1H), 8.38 (d, J = 5.3 Hz, 1H), 8.31 (dd, J = 8.6, 0.9 Hz, 1H), 8.20 (dd, J = 8.5, 0.9 Hz, 1H), 7.79-7.76 (m, 2H), 7.62-7.58 (m, 2H), 7.44 (ddd, J = 8.3, 6.9, 1.4 Hz, 1H), 7.41 (ddd, J = 8.3, 6.9, 1.4 Hz, 1H), 7.29 (br t, J = 5.2 Hz, 1H), 6.79 (br d, J = 7.6 Hz, 1H), 6.51 (d, J = 5.6 Hz, 1H), 6.48 (d, J = 5.4 Hz, 1H), 4.03 (q, J = 7.1 Hz, trace ethyl acetate), 3.51 (m, 1H), 3.33 (m, partly buried under water signal, approximately 2H), 2.97 (m, 2H), 2.46 (t, J = 6.8 Hz, 2H), 2.11-2.07 (m, 2H), 2.01-2.00 (m, 2H), 1.99 (s, trace ethyl acetate), 1.85 (m, 2H), 1.69 (m, 2H), 1.18 (t, J = 7.1 Hz, trace ethyl acetate).

^{13}C NMR δ (ppm)(DMSO-d6): 170.7 (trace ethyl acetate), 150.7, 150.7, 150.0, 149.0, 148.5, 148.3, 129.1, 129.0, 128.7, 123.8, 123.6, 122.0, 121.6, 118.9, 118.8, 98.6, 98.1, 59.7 (trace ethyl acetate) 55.8, 52.4, 49.5, 41.0, 31.1, 25.4, 20.8 (trace ethyl acetate), 14.1 (trace ethyl acetate). *Note: One aromatic resonance was not observed in this spectrum. This was presumed to be due to low signal to noise.*

^{1}H NMR δ (ppm)($CDCl_3$): 8.56-8.58 (2 H, m), 7.95-8.04 (2 H, m), 7.87 (1 H, dd, J = 8.38, 1.31 Hz), 7.77 (1 H, dd, J = 8.41, 1.25 Hz), 7.65-7.65 (2 H, m), 7.49-7.49 (1 H, m), 7.41-7.41 (1 H, m), 7.01 (1 H, br t, J = 4.25 Hz), 6.49 (1 H, d, J = 5.35 Hz), 6.40 (1 H, d, J = 5.34 Hz), 4.95 (1 H, br d, J = 6.91 Hz), 3.66-3.66 (1 H, m), 3.43 (2 H, td, J = 6.02, 4.41 Hz), 3.04-3.10 (2 H, m), 2.66-2.68 (2 H, m), 2.22-2.35 (4 H, m), 1.99 (2 H, m), 1.77-1.80 (2 H, m).

MS (ESI): *m/z* 412.25005 M + H (calculated 412.24957)

HPLC (method A) t_R = 4.06 min (97% pure).

Compound 9: 7-chloro-*N*-(1-(3-((8-(trifluoromethyl)quinolin-4-yl)amino)propyl)piperidin-4-yl)quinolin-4-amine (Burgess, 2010)

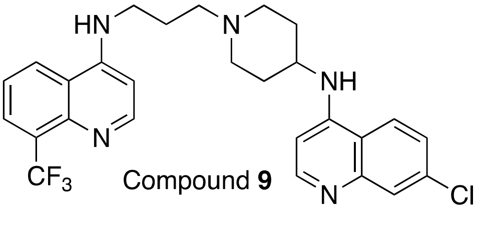

Compound **9**

3-(8-(Trifluoromethyl)quinolin-4-ylamino)propyl methanesulfonate (0.50 g, 0.00144 mol), 7-chloro-*N*-(piperidin-4-yl)quinolin-4-amine (0.39 g, 0.00151 mol), K_2CO_3 (0.20 g, 0.00145 mol), and a catalytic amount of potassium iodide were heated for 5 days in refluxing acetonitrile (40 mL), at which point TLC indicated that reaction was complete. After dilution with 10 mL water, the acetonitrile was removed by evaporation under reduced pressure with warming, and the residue was combined with chloroform (30 mL) and water (30 mL). A beige solid remained undissolved and was removed by filtration, rinsing with chloroform and water. After separation of the chloroform and water layers, the aqueous layer was extracted with chloroform (3 x 7 mL), and the pooled organic layers were dried ($MgSO_4$) and evaporated under reduced pressure with warming to yield a pale yellow, waxy solid, 0.09g. NMR indicated that the solid from filtration was primarily the desired product. This was dissolved in excess boiling 95% ethanol, and allowed to evaporate gradually (at room temperature) to approximately 10 mL. Filtration afforded the desired product as sparkling, beige crystals (0.52 g, 70%, mp = 225-233°C (dec)).

^{1}H NMR δ (ppm)(DMSO-d_6): 8.49-8.52 (2 H, m), 8.36-8.39 (2 H, m), 8.03 (1 H, d, J = 7.29 Hz), 7.78 (1 H, d, J = 2.25 Hz), 7.53-7.57 (2 H, m), 7.45 (1 H, dd, J = 9.01, 2.28 Hz), 6.98 (1 H, br d, J = 7.48 Hz), 6.62 (1 H, d, J = 5.50 Hz), 6.55 (1 H, d, J = 5.57 Hz), 3.44-3.53 (1 H, m), 3.35-3.36 (2 H, m), 2.96 (2 H, m), 2.44-2.47 (2 H, m), 2.09 (2 H, t, J = 11.56 Hz), 1.99 (2 H, m), 1.86 (2 H, m), 1.62-1.71 (2 H, m).

^{13}C NMR δ (ppm)(DMSO-d_6): 151.9, 151.4, 150.3, 149.3, 149.1, 144.9, 133.4, 127.4, 126.7, 124.4, 123.9, 123.1, 122.4, 119.4, 117.5, 99.1, 99.0, 55.6, 52.3, 49.6, 40.9, 31.0, 25.3.

MS (ESI): *m/z* 514.19866 M + H (calculated 514.19798)

HPLC (method A) t_R = 6.53 min (99% pure).

Compound 10: 8-(trifluoromethyl)-*N*-(3-(4-((8-(trifluoromethyl)quinolin-4-yl)amino)piperidin-1-yl)propyl)quinolin-4-amine (Burgess, 2010)

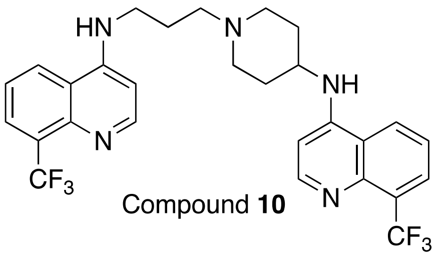

Compound **10**

3-(8-(Trifluoromethyl)quinolin-4-ylamino)propyl methanesulfonate (0.66 g, 1.9 mmol), *N*-(piperidin-4-yl)-8-(trifluoromethyl)quinolin-4-amine (0.59 g, 2.0 mmol), K_2CO_3 (0.39 g, 2.8 mmol), and a catalytic amount of potassium iodide were heated for 27 hours in refluxing acetonitrile (50 mL), at which point TLC indicated that reaction was complete; however, heating at reflux was continued for an additional 18 hours. The solvent was removed by evaporation under reduced pressure with warming, and the resulting golden brown solid was combined with chloroform (30 mL) and water (30 mL). A large amount of solid material remained undissolved and was removed by filtration, rinsing with chloroform and water (a white solid). This material was dissolved in excess boiling ethyl acetate, followed by gradual evaporation at room temperature to induce crystallization. The fine, off-white crystals thus obtained proved to be the desired product (0.20 g, 19%).

^{1}H NMR δ (ppm)($CDCl_3$): 8.72 (1 H, d, J = 5.35 Hz), 8.69 (1 H, d, J = 5.35 Hz), 8.03-8.07 (2 H, m), 7.96-8.00 (2 H, m), 7.52 (1 H, dd, J = 8.41, 7.33 Hz), 7.42 (1 H, dd, J = 8.41, 7.27 Hz), 7.09 (1 H, br t, J = 4.24 Hz), 6.58 (1 H, d, J = 5.41 Hz), 6.48 (1 H, d, J = 5.41 Hz), 4.97 (1 H, br d, J = 6.71 Hz), 3.66-3.69 (1 H, m), 3.44 (2 H, td, J = 6.01, 4.30 Hz), 3.06-3.09 (2 H, m), 2.68 (2 H, t, J = 5.59 Hz), 2.28-2.33 (4 H, m), 1.99-2.05 (2 H, m), 1.73-1.82 (2 H, m).

^{13}C NMR δ (ppm)($CDCl_3$): 152.0, 151.7, 150.4, 148.3, 145.6, 145.5, 127.7, 127.4, 124.6, 123.5, 123.2, 122.3, 119.8, 119.4, 100.1, 99.3, 58.2, 52.5, 43.9, 32.0, 24.3.

MS (ESI): *m*/*z* 548.22434 M + H (calculated 548.22519)

HPLC (method A) t_R = 6.93 min (99% pure).

Compound 8: 7-chloro-*N*-(3-(4-((8-(trifluoromethyl)quinolin-4-yl)amino)piperidin-1-yl)propyl)quinolin-4-amine (Burgess, 2010)

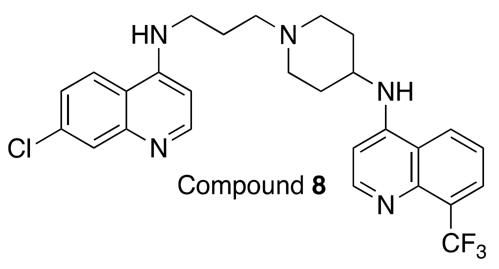

Compound **8**

3-(7-Chloroquinolin-4-ylamino)propyl methanesulfonate (0.59 g, 1.9 mmol), *N*-(piperidin-4-yl)-8-(trifluoromethyl)quinolin-4-amine (0.58 g, 2.0 mmol), K_2CO_3 (0.39 g, 2.8 mmol), and a catalytic amount of potassium iodide were heated for 24 hours in refluxing acetonitrile (50 mL), at which point TLC indicated that reaction was complete. The solvent was removed by evaporation under reduced pressure with warming, and the resulting ochre solid was combined with chloroform (30 mL) and water (30 mL). A large amount of solid material remained undissolved and was removed by filtration, rinsing with chloroform and water. The chloroform and water layers of the filtrate were separated, and the aqueous layer was extracted with chloroform (3 x 10 mL), dried ($MgSO_4$), and evaporated under reduced pressure with warming. This material was combined with the insoluble material from extraction (above), and taken up in excess boiling 95% ethanol. This was allowed to cool and evaporate gradually at room temperature. The crystals that formed were recovered by filtration and further purified by automated flash chromatography on basic alumina, eluting with a gradient of 100% hexanes to 100% ethyl acetate. The desired product was obtained as a beige solid (0.38 g, 40%, mp = 205-207°C, R_f = 0.2 (alumina, EA)).

^{1}H NMR δ (ppm)($CDCl_3$): 8.72 (1 H, d, J = 5.35 Hz), 8.54 (1 H, d, J = 5.35 Hz), 8.04 (1 H, d, J = 7.31 Hz), 7.99 (1 H, d, J = 8.53 Hz), 7.97 (1 H, d, J = 2.15 Hz), 7.78 (1 H, d, J = 8.92 Hz), 7.53 (1 H, dd, J = 8.44, 7.30 Hz), 7.35 (1 H, dd, J = 8.88, 2.17 Hz), 7.03 (1 H, br t, J = 4.28 Hz), 6.58 (1 H, d, J = 5.41 Hz), 6.38 (1 H, d, J = 5.39 Hz), 4.98 (1 H, br d, J = 6.79 Hz), 3.66-3.68 (1 H, m), 3.43 (2 H, td, J = 5.99, 4.32 Hz), 3.06-3.09 (2 H, m), 2.68 (2 H, t, J = 5.59 Hz), 2.28-2.35 (4 H, m), 1.99 (2 H, m), 1.71-1.80 (2 H, m).

^{13}C NMR δ (ppm)(DMSO-d_6): 151.9, 151.3, 150.1, 149.3, 149.0, 145.0, 133.3, 127.4, 127.1, 125.8, 124.0, 123.9, 122.1, 119.3, 117.4, 99.5, 98.5, 55.6, 52.3, 49.6, 40.9, 30.9, 25.3.

MS (ESI): *m*/*z* 514.19879 M + H (calculated 514.19798)

HPLC (method A) t_R = 6.95 min (>99% pure).

Compound 6: 7-chloro-*N*-(3-(4-((7-chloroquinolin-4-yl)amino)piperidin-1-yl)propyl)quinolin-4-amine (Burgess, 2010)

Preferred method:

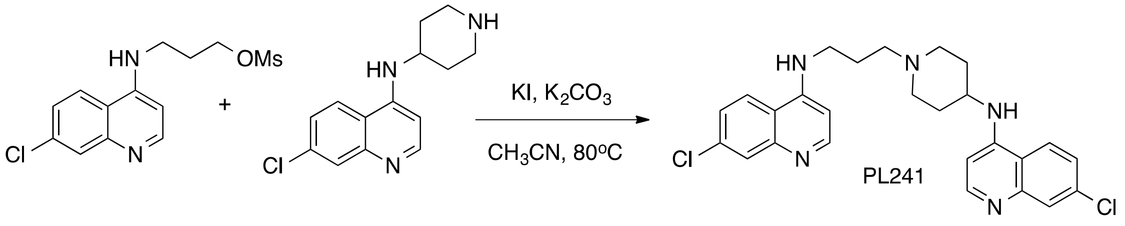

3-(7-Chloroquinolin-4-ylamino)propyl methanesulfonate (1.20 g, 3.8 mmol), 7-chloro-*N*-(piperidin-4-yl)quinolin-4-amine (1.05 g, 4.0 mmol), potassium carbonate (5.7 mmol, 0.79 g), a catalytic amount of potassium iodide, and 50 mL anhydrous acetonitrile were allowed to heat for 48 hours at reflux, whereupon TLC indicated that the reaction was complete. The reaction mixture was diluted with water (50 mL) and vacuum filtered. The filtrate was concentrated under reduced pressure with warming, and the reaction mixture was partitioned between 50/50 dichloromethane/chloroform (20 mL) and 10 mL saturated sodium bicarbonate, followed by further extraction with three 10 mL portions dichloromethane. The pooled organic layers were dried over anhydrous magnesium sulfate and concentrated under reduced pressure. The resulting solid was combined with the material filtered from the reaction mixture and recrystallized from 95% ethanol, which afforded the desired product as a pale yellow, crystalline solid (1.30 g). Concentration of the mother liquor yielded a further crop of crystals (0.06 g, total yield 71%, mp = 224-227°C (dec)).

^{1}H NMR δ (ppm)($CDCl_3$): 8.56 (1 H, d, J = 5.33 Hz, Q_1-C2-H), 8.53 (1 H, d, J = 5.33 Hz, Q_2-C2-H), 7.99 (1 H, d, J = 2.16 Hz, Q_1-C5-H), 7.97 (1 H, d, J = 2.14 Hz, Q_2-C5-H), 7.78 (1 H, d, J = 8.90 Hz, Q_2-C8-H), 7.71 (1 H, d, J = 8.95 Hz, Q_1-C8-H), 7.43 (1 H, dd, J = 8.89, 2.18 Hz, Q_1-C6-H), 7.35 (1 H, dd, J = 8.87, 2.16 Hz, Q_2-C6-H), 7.02 (1 H, br t, J = 4.27 Hz, Q_1-C4-N<u>H</u>), 6.47 (1 H, d, J = 5.37 Hz, Q_1-C3-H), 6.38 (1 H, d, J = 5.37 Hz, Q_2-C3-H), 4.92 (1 H,

br d, J = 6.78 Hz, Q_2-N<u>H</u>), 3.65 (1 H, m, Pip-C<u>H</u>), 3.42 (2 H, td, J_{CH2} = 6.01, J_{NH} = 4.35 Hz, Q_1-NHC<u>H</u>$_2$CH$_2$CH$_2$), 3.06 (2 H, m, piperidine-CH x 2 adjacent to alkyl chain), 2.67 (2 H, t, J_{CH2} = 5.64 Hz, Q_1-NHCH$_2$CH$_2$C<u>H</u>$_2$), 2.33 (2 H, m, piperidine-CH x 2 adjacent to alkyl chain), 2.28 (2 H, m, piperidine CH x 2 adjacent to CH-NH-Q_2), 1.99 (2 H, m, Q_1-NHCH$_2$C<u>H</u>$_2$CH$_2$), 1.75 (water signal overlaps m, ~2 H, piperidine CH x 2 adjacent to CH-NH-Q_2).

^{13}C NMR δ (ppm)($CDCl_3$): 152.3 (Q_1-C2), 152.0 (Q_2-C2), 150.4, 149.4, 149.3, 148.4, 135.1, 134.7, 129.1 (Q-C5), 129.0 (Q-C5), 125.6 (Q_1-C6), 124.8 (Q_2-C6), 121.7 (Q_2-C8), 120.7 (Q_1-C8), 117.5, 117.2, 99.6 (Q_1-C3), 98.8 (Q_2-C3), 58.2 (Q_1-NHCH$_2$CH$_2$<u>C</u>H$_2$), 52.5 (piperidine-C adjacent to alkyl chain), 49.5 (piperidine-<u>C</u>H-NH- Q_2), 43.9 (Q_1-NH<u>C</u>H$_2$CH$_2$CH$_2$), 32.0 (piperidine-C adjacent to CH-NH-Q_2), 24.4 (Q_1-NHCH$_2$<u>C</u>H$_2$CH$_2$).

*Note: Q_1 and Q_2 denote respectively the quinoline ring system on the left and that on the right of the structure as shown above. Spectra are provided below (Example spectra: Compound **6**).*

MS (ESI): *m/z* 480.17456 M + H (calculated 480.17163)

HPLC (method A) t_R = 6.93 min (97% pure).

Original method used to synthesize compound 6 (for preferred method, see above)

***N*-(3-(4-aminopiperidin-1-yl)propyl)-7-chloroquinolin-4-amine**:

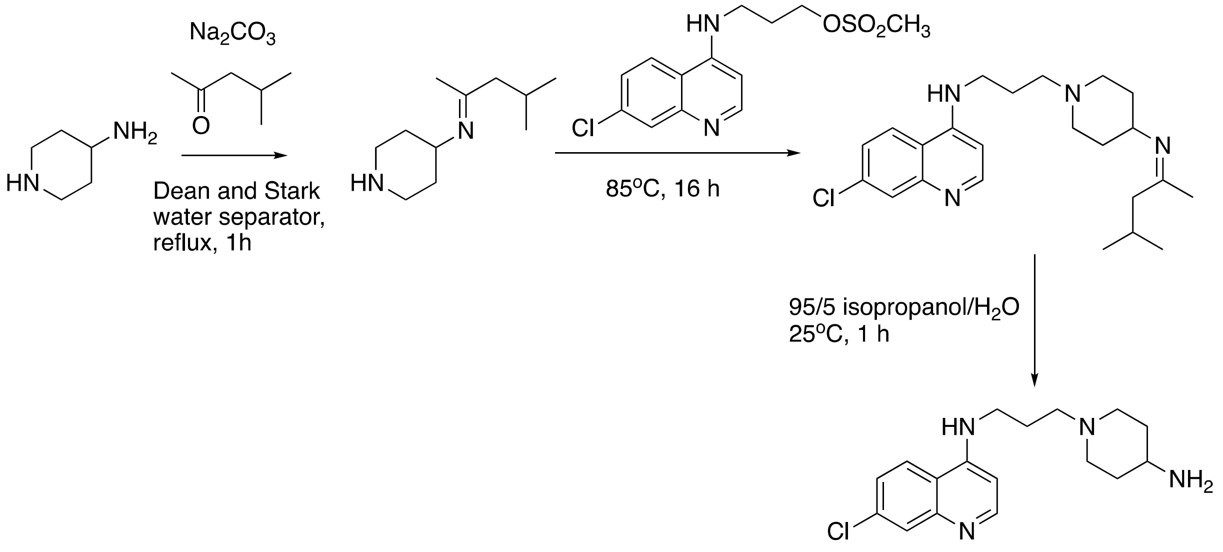

Methyl isobutyl ketone, 125 mL, was allowed to reflux in a round bottom flask equipped with a Dean and Stark water separator. After 30 minutes, sodium carbonate (0.74 g, 7.0 mmol) and 4-aminopiperidine (0.580 mL, 5.5 mmol) were added and reflux was continued for 1 hour, at the end of which time TLC indicated that no more of the amine starting material was present. The reaction was allowed to cool to 85°C, and 3-(7-chloroquinolin-4-ylamino)propyl methanesulfonate (1.45 g, 4.6 mmol) was added. After 16 hours had elapsed, TLC indicated that no more 3-(7-chloroquinolin-4-ylamino)propyl methanesulfonate was present. The reaction mixture was washed with water (50 mL) to remove sodium carbonate, and the solvent was then evaporated under reduced pressure. The resulting amber oil was stirred for 1.5 hours in a mixture of 50 mL isopropanol and 5 mL water, and the solvent was then evaporated under reduced pressure. The resulting liquid was partitioned between ethyl acetate (20 mL) and saturated sodium bicarbonate (25 mL), and the aqueous layer was extracted with additional ethyl acetate (3 x 10 mL). During this procedure, a yellow oil remained insoluble; NMR indicated that this was pure product. After removal of water therefrom by heating under reduced pressure, this material was used without further purification.

Compound 6, original method, final step (Andersag, 1948; Surrey, 1951):

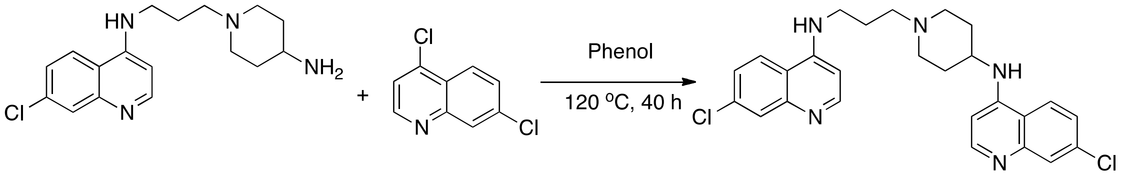

N-(3-(4-Aminopiperidin-1-yl)propyl)-7-chloroquinolin-4-amine (0.30 g, approximately 0.94 mmol), 4,7-dichloroquinoline (0.59g, 3.00 mmol), and 1.3 g phenol were heated for 40 hours at 120°C. The resulting dark brown oil was taken up in dichloromethane (20 mL) and washed with 1.0 M caustic soda (6 x 10 mL), followed by brine (10 mL). The organic layer was dried over anhydrous magnesium sulfate and evaporated under reduced pressure to yield a brown oil. This was purified by column chromatography on alumina, eluting with ethyl acetate and then with 95/5 ethyl acetate/methanol (v/v). The resulting solid was recrystallized using a Craig tube from 4:1 ethyl

acetate:methanol, which afforded the desired product as a beige solid (0.034g, 7%, R_f = 0.6 (basic alumina, EA/MeOH, 85/15, v/v)).

<u>Sample NMR spectra: Compound 6</u>

The following spectra were run at 600 MHz. The solvent used was $CDCl_3$, except where noted (it was necessary to run an additional ^1H spectrum in DMSO-d6 to confirm the suspected integration of one peak that was overlapped by the water signal in $CDCl_3$). TMS was used as a reference. The one-dimensional spectra shown here were processed using DataChord Spectrum Analyst 4.1.rc39, while the two-dimensional spectra were processed using Bruker Topspin 2.1.

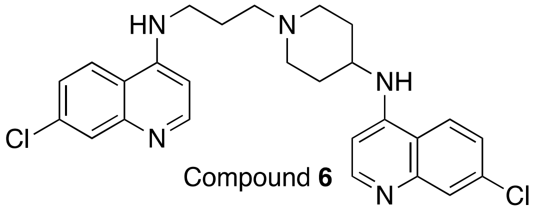

Compound **6**

Spectral assignments:

^1H NMR δ (ppm)($CDCl_3$): 8.56 (1 H, d, J = 5.33 Hz, Q_1-C2-H), 8.53 (1 H, d, J = 5.33 Hz, Q_2-C2-H), 7.99 (1 H, d, J = 2.16 Hz, Q_1-C8-H), 7.97 (1 H, d, J = 2.14 Hz, Q_2-C8-H), 7.78 (1 H, d, J = 8.90 Hz, Q_2-C5-H), 7.71 (1 H, d, J = 8.95 Hz, Q_1-C5-H), 7.43 (1 H, dd, J = 8.89, 2.18 Hz, Q_1-C6-H), 7.35 (1 H, dd, J = 8.87, 2.16 Hz, Q_2-C6-H), 7.02 (1 H, br t, J = 4.27 Hz, Q_1-C4-N$\underline{H}$), 6.47 (1 H, d, J = 5.37 Hz, Q_1-C3-H), 6.38 (1 H, d, J = 5.37 Hz, Q_2-C3-H), 4.92 (1 H, br d, J = 6.78 Hz, Q_2-N$\underline{H}$), 3.65 (1 H, m, Pip-C$\underline{H}$), 3.42 (2 H, td, J_{CH2} = 6.01, J_{NH} = 4.35 Hz, Q_1-NHC$\underline{H}_2$$CH_2CH_2$), 3.06 (2 H, m, piperidine-CH x 2 adjacent to alkyl chain), 2.67 (2 H, t, J_{CH2} = 5.64 Hz, Q_1-NHCH_2CH_2C$\underline{H}_2$), 2.33 (2 H, m, piperidine-CH x 2 adjacent to alkyl chain), 2.28 (2 H, m, piperidine CH x 2 adjacent to CH-NH-Q_2), 1.99 (2 H, m, Q_1-NHCH_2C$\underline{H}_2$$CH_2$), 1.75 (water signal overlaps m, ~2 H, piperidine CH x 2 adjacent to CH-NH-Q_2).

^{13}C NMR δ (ppm)($CDCl_3$): 152.3 (Q_1-C2), 152.0 (Q_2-C2), 150.4, 149.4, 149.3, 148.4, 135.1, 134.7, 129.1 (Q-C8), 129.0 (Q-C8), 125.6 (Q_1-C6), 124.8 (Q_2-C6), 121.7 (Q_2-C5), 120.7 (Q_1-C5), 117.5, 117.2, 99.6 (Q_1-C3), 98.8 (Q_2-C3), 58.2 (Q_1-NH$CH_2CH_2$$\underline{C}H_2$), 52.5 (piperidine-C

adjacent to alkyl chain), 49.5 (piperidine-C̲H-NH- Q_2), 43.9 (Q_1-NHC̲$H_2CH_2CH_2$), 32.0 (piperidine-C adjacent to CH-NH-Q_2), 24.4 (Q_1-NHCH_2C̲H_2CH_2).

Note: Q_1 and Q_2 denote the quinoline ring system on the left and right of the figure, respectively.

List of spectra:

Figure C1: ^{1}H NMR spectrum of compound **6** in $CDCl_3$, full view.
Figure C2: ^{1}H NMR spectrum of compound **6** in $CDCl_3$, expansion 1 of 3 (aromatic region, part 1).
Figure C3: ^{1}H NMR spectrum of compound **6** in $CDCl_3$, expansion 2 of 3 (aromatic region, part 2).
Figure C4: ^{1}H NMR spectrum of compound **6** in $CDCl_3$, expansion 3 of 3 (aliphatic region, part 1).
Figure C5: ^{13}C NMR spectrum of compound **6** in $CDCl_3$, full view.
Figure C6: ^{13}C NMR spectrum of compound **6** in $CDCl_3$, aliphatic region only.
Figure C7: COSY spectrum of compound **6** in $CDCl_3$.
Figure C8: HSQC spectrum of compound **6** in $CDCl_3$.
Figure C9: HMBC spectrum of compound **6** in $CDCl_3$.
Figure C10: NOESY spectrum of compound **6** in $CDCl_3$.
Figure C11: ^{1}H NMR spectrum of compound **6** in DMSO-d6.
Figure C12: ^{1}H NMR spectrum of compound **6** in DMSO-d6, aliphatic region only.

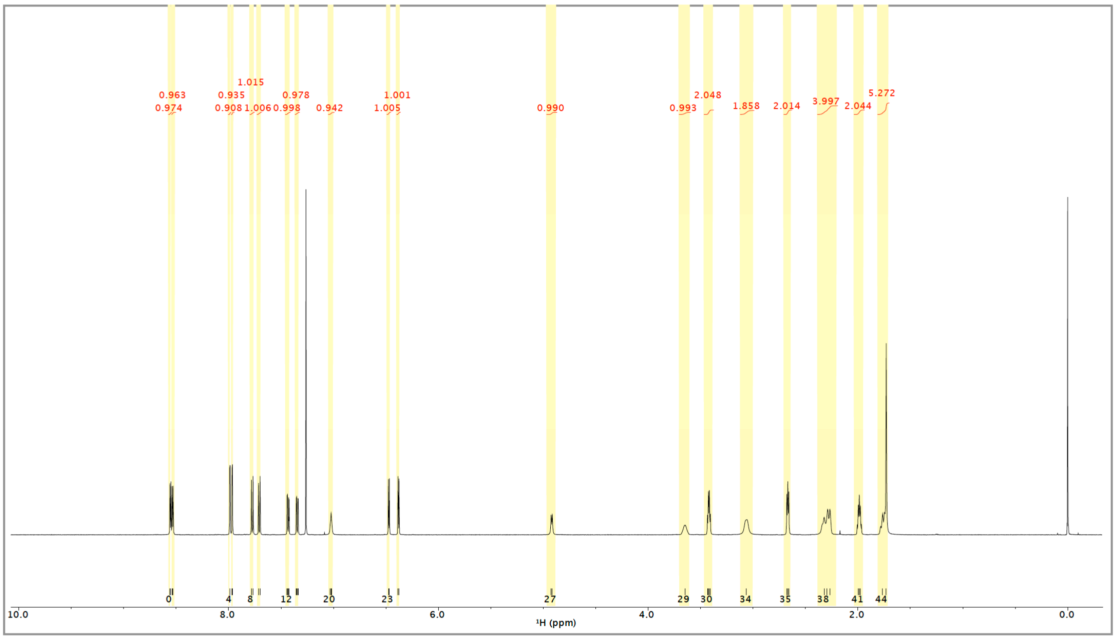

Figure C1: ^{1}H NMR spectrum of compound **6** in $CDCl_3$, with integrals (see figures C2, C3, and C4 for expanded views).

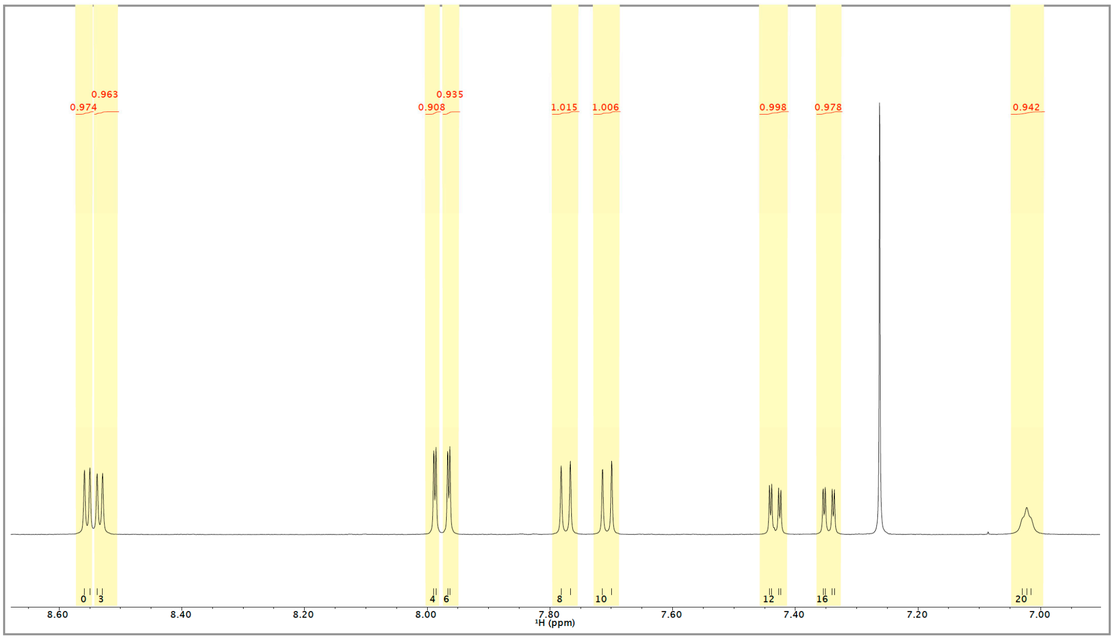

6

Figure C2: ^{1}H NMR spectrum of compound **6** in $CDCl_3$, expansion 1 of 3 (aromatic region, part 1).

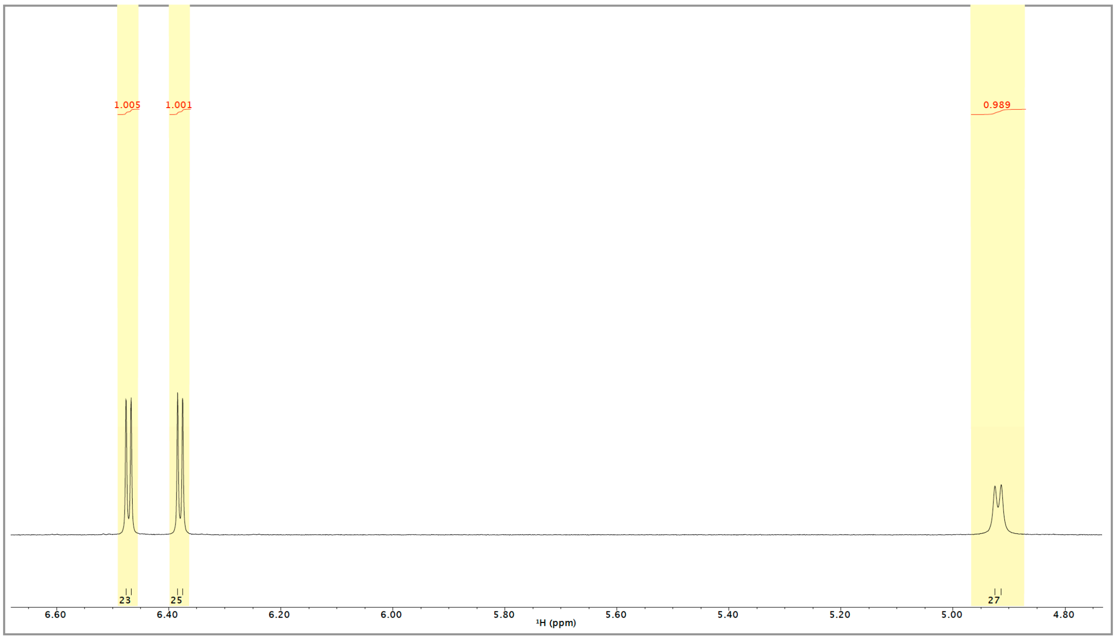

Figure C3: ^{1}H NMR spectrum of compound **6** in $CDCl_3$, expansion 2 of 3 (aromatic region, part 2).

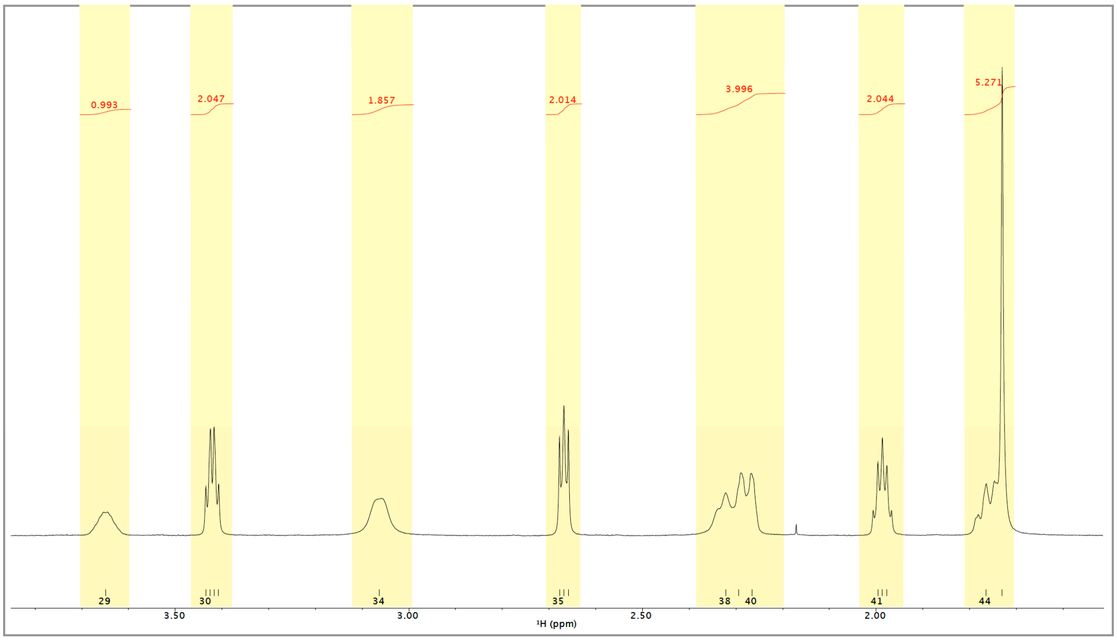

Figure C4: ^{1}H NMR spectrum of compound **6** in $CDCl_3$, expansion 3 of 3 (aliphatic region).

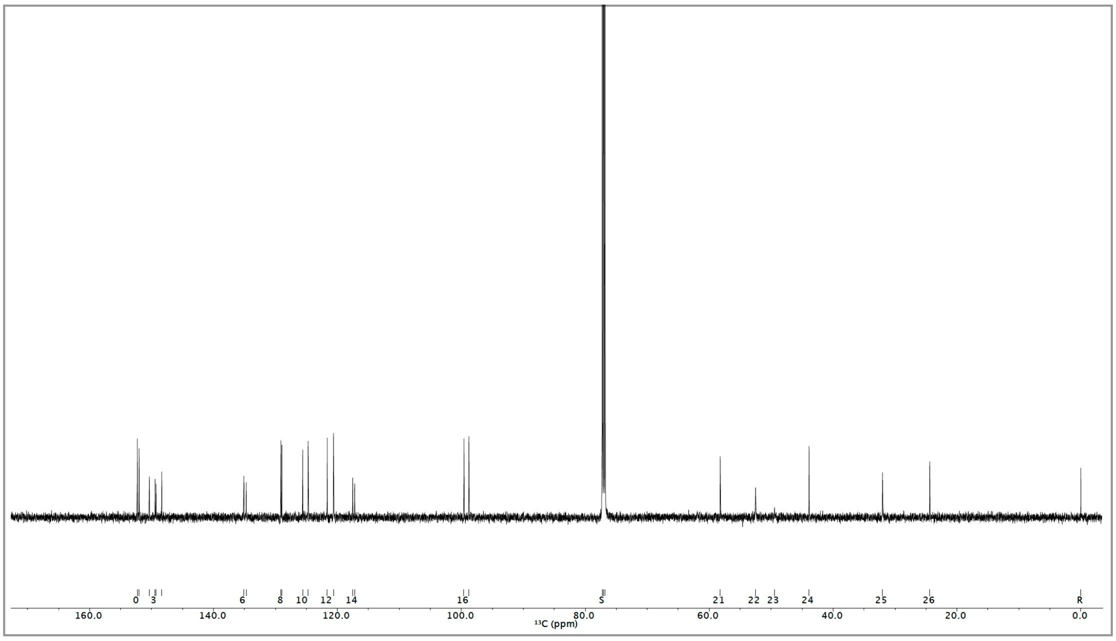

Figure C5: ^{13}C NMR spectrum of compound **6** in $CDCl_3$ (see figure C6 for expanded aromatic region).

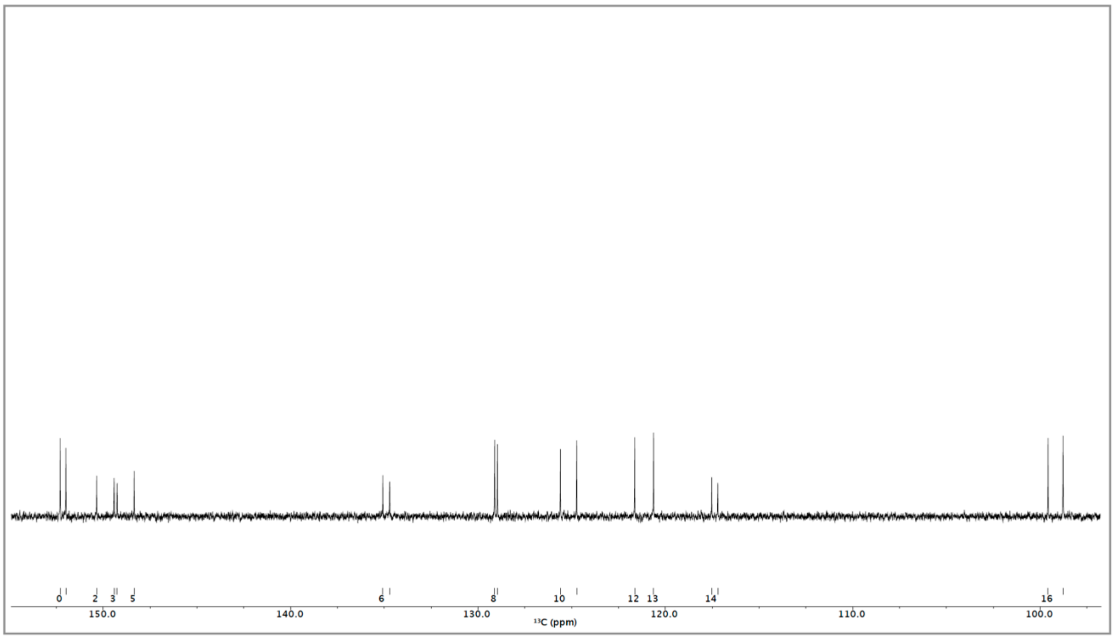

Figure C6: ^{13}C NMR spectrum of compound **6** in $CDCl_3$, aromatic region only.

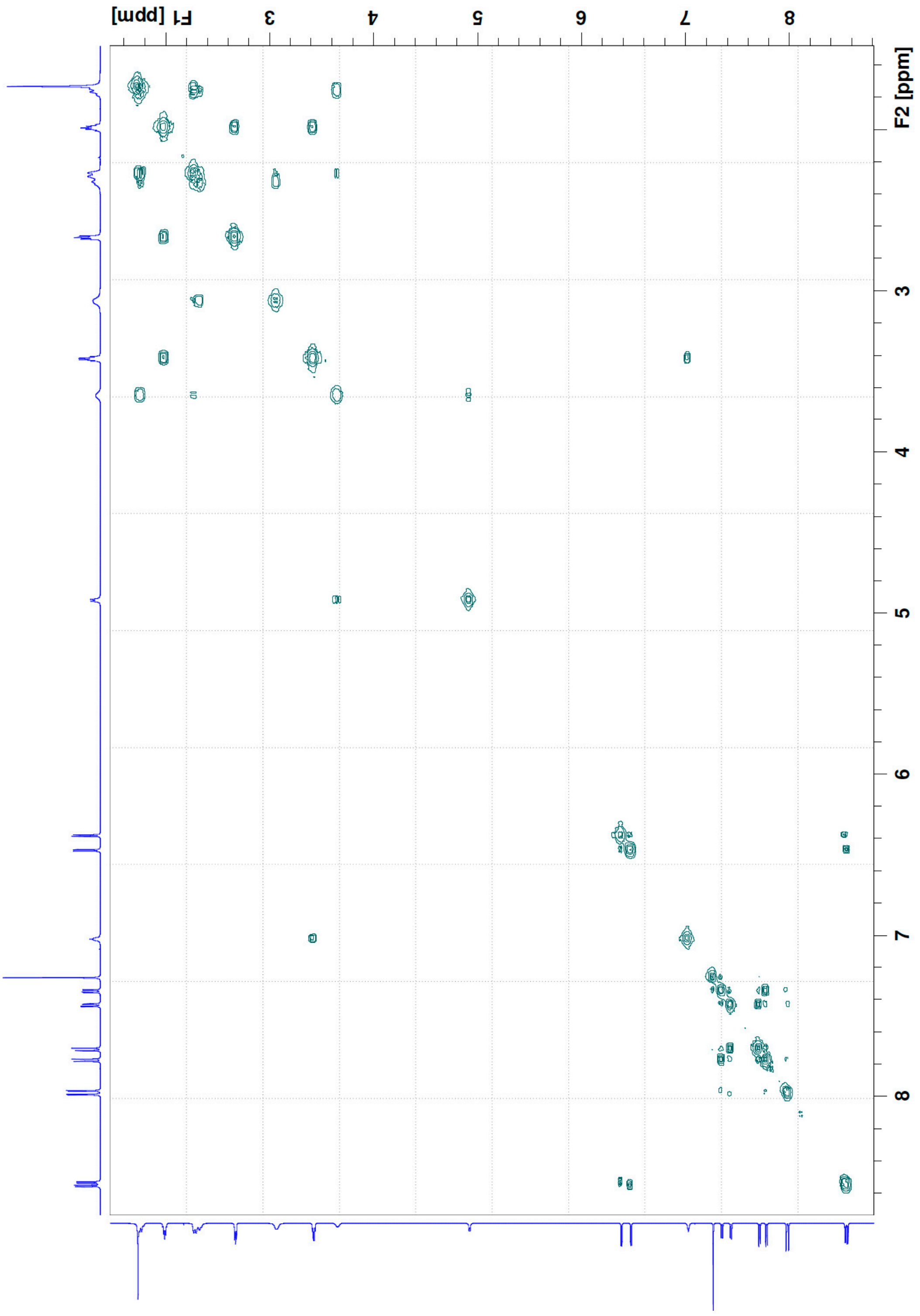

Figure C7: COSY spectrum of compound **6** in $CDCl_3$, showing direct proton to proton interactions.

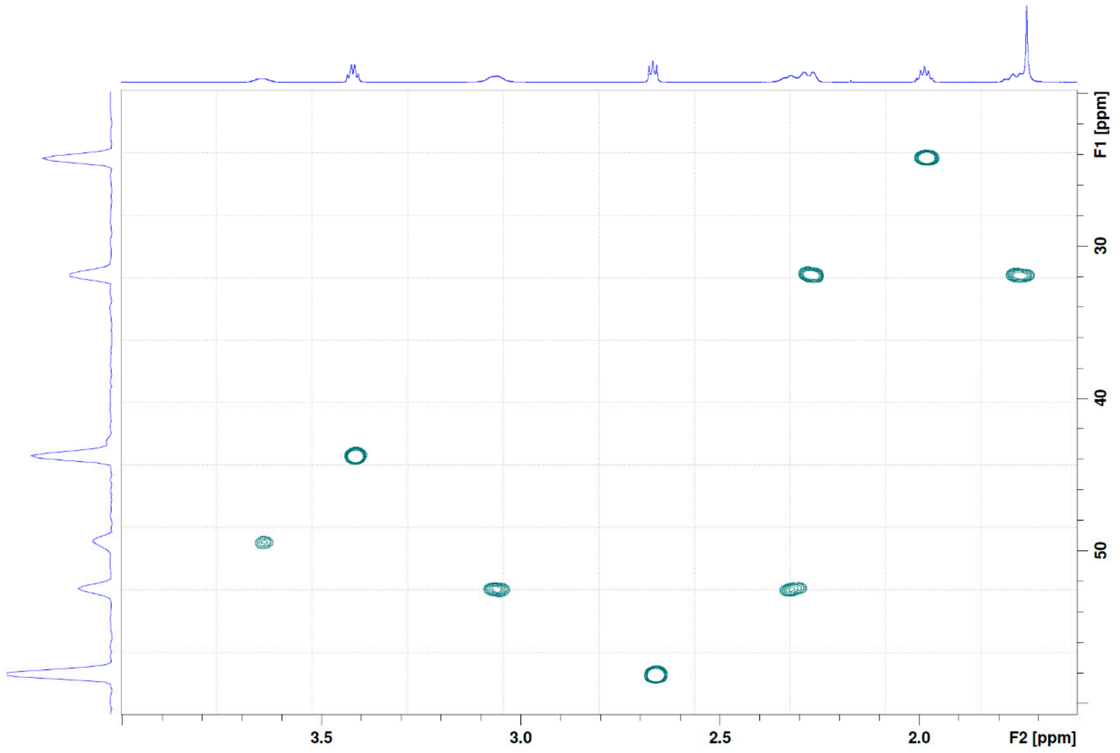

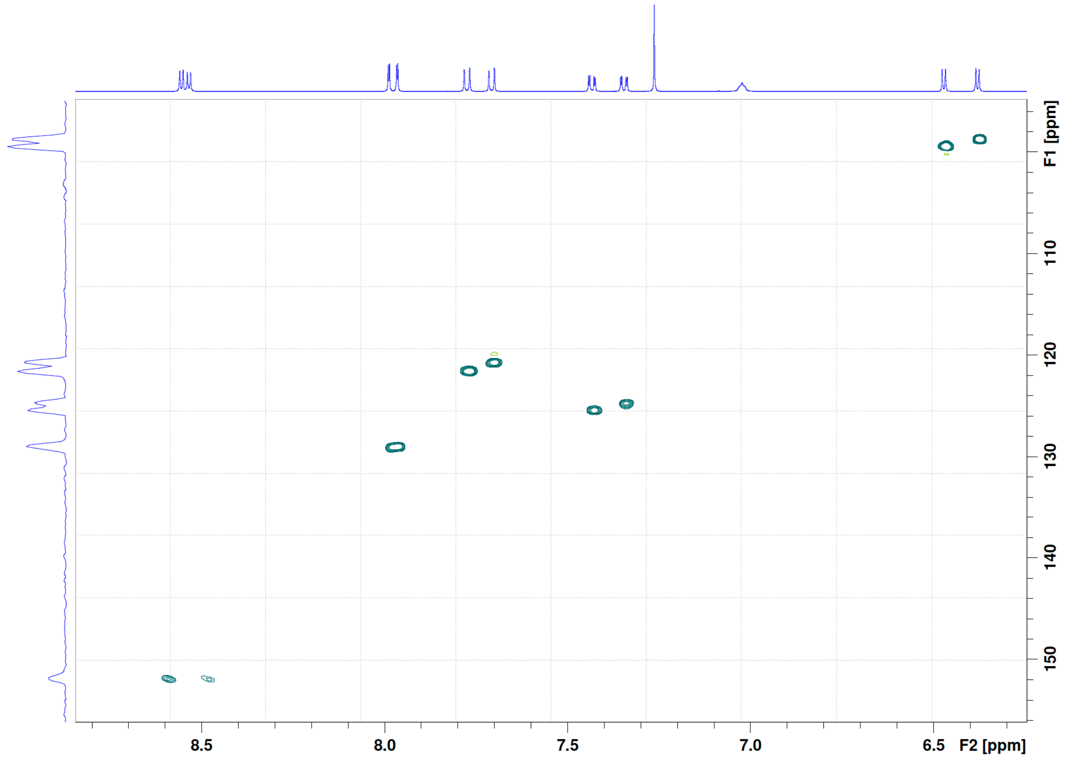

Figure C8: HSQC spectrum of compound **6** in $CDCl_3$, showing direct proton to carbon interactions (split into two expanded regions to show detail).

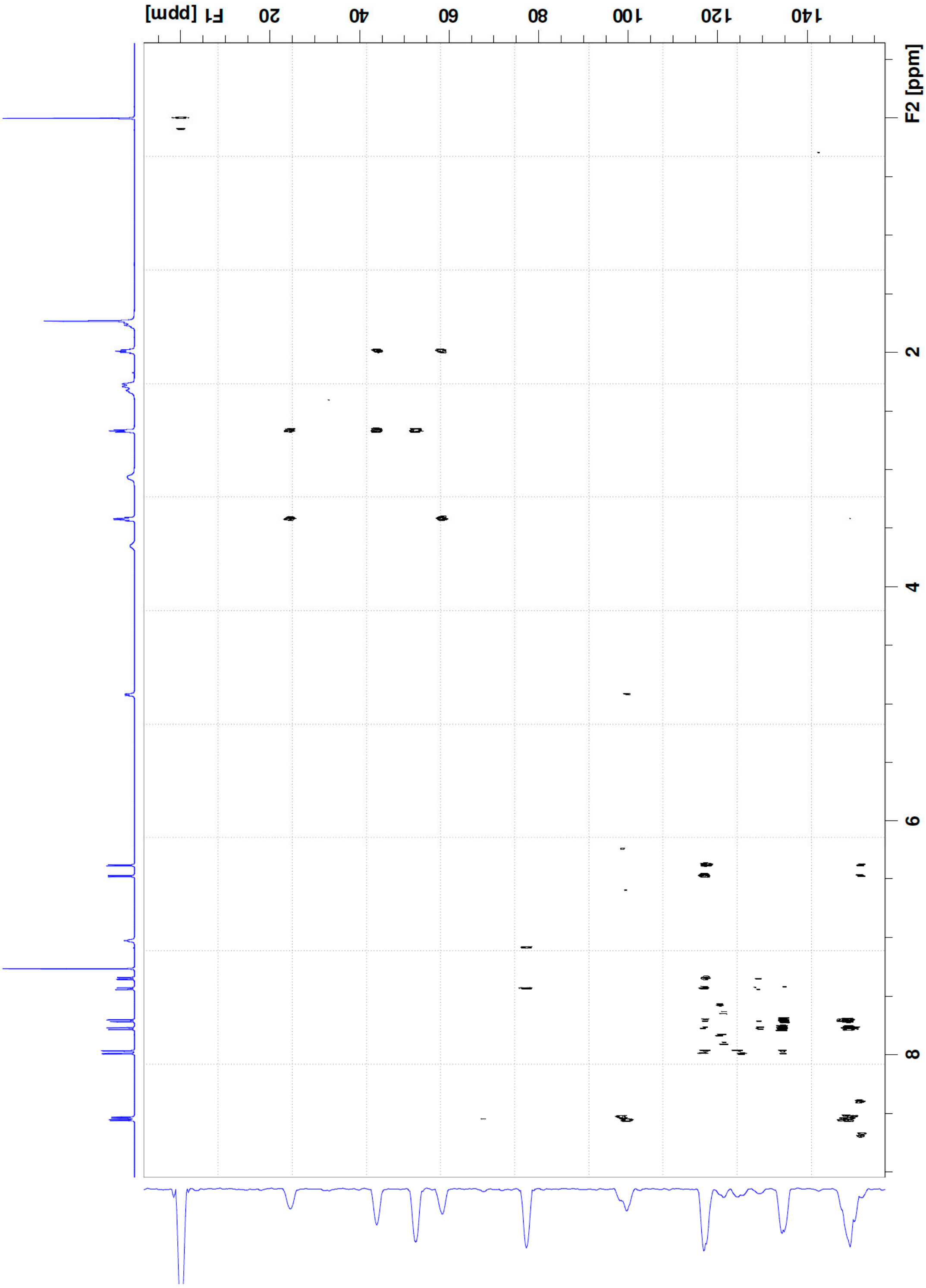

Figure C9: HMBC spectrum of compound **6** in $CDCl_3$, showing ^{13}C - ^{1}H multiple bond correlations.

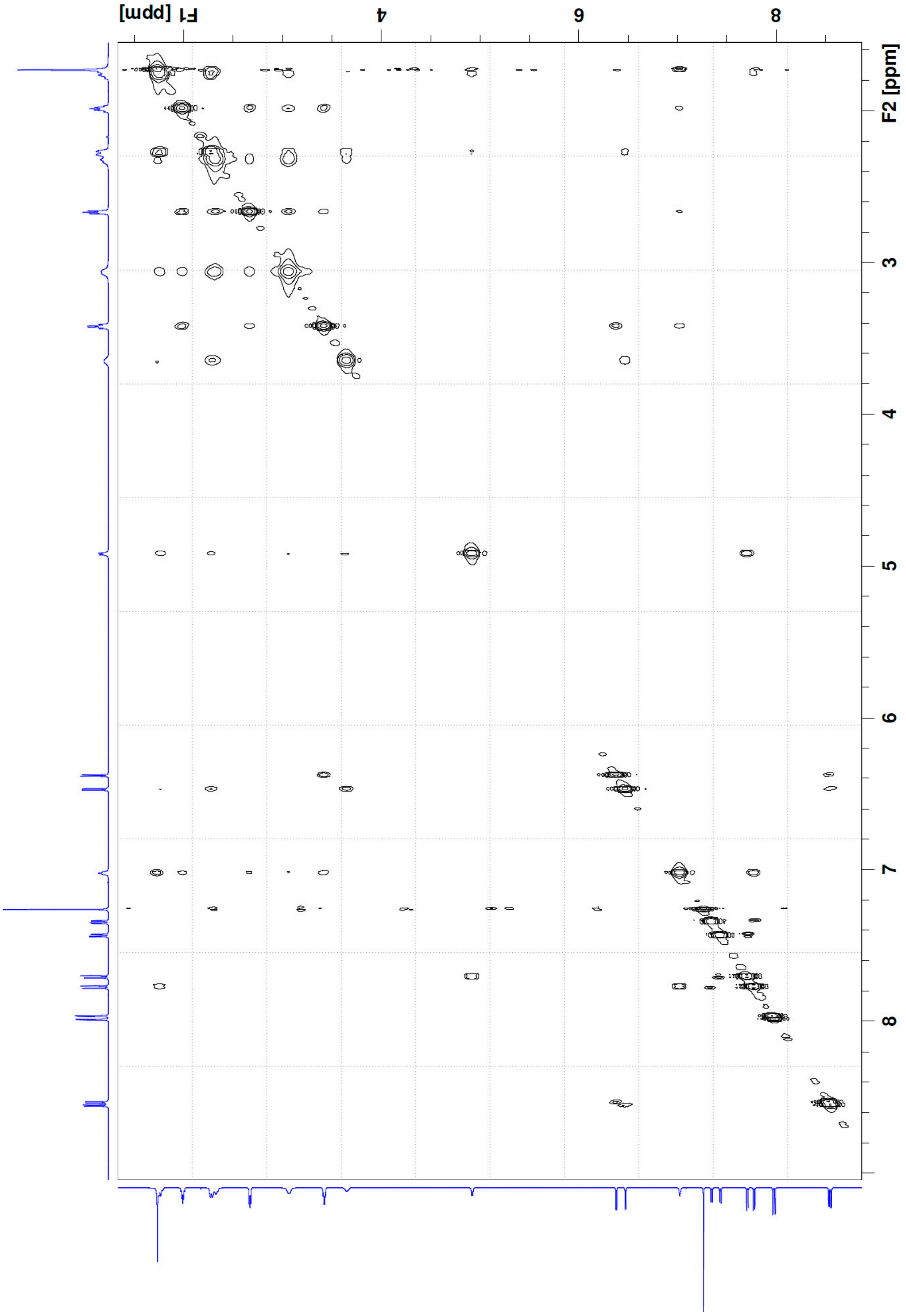

Figure C10: NOESY spectrum of compound **6** in $CDCl_3$, showing proton to proton interactions through space.

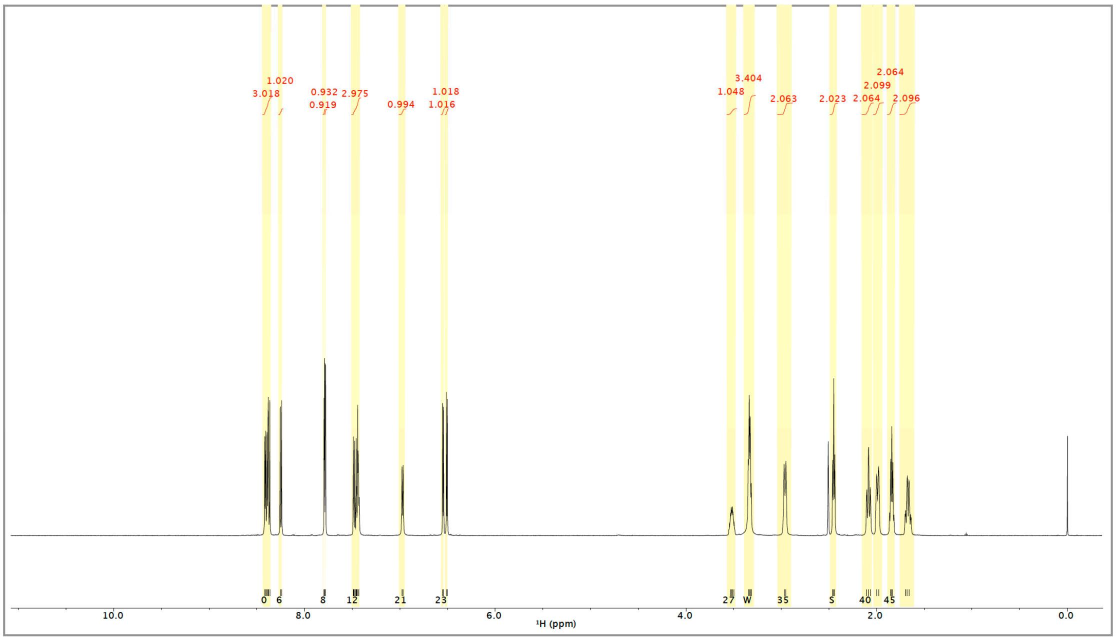

Figure C11: ^{1}H NMR spectrum of compound **6** in DMSO-d6, with integrals (see figure C12 for expanded aliphatic region). This spectrum was used to confirm the suspected integration of a peak overlapped by the water signal in the $CDCl_3$ spectrum (here at 1.68 ppm).

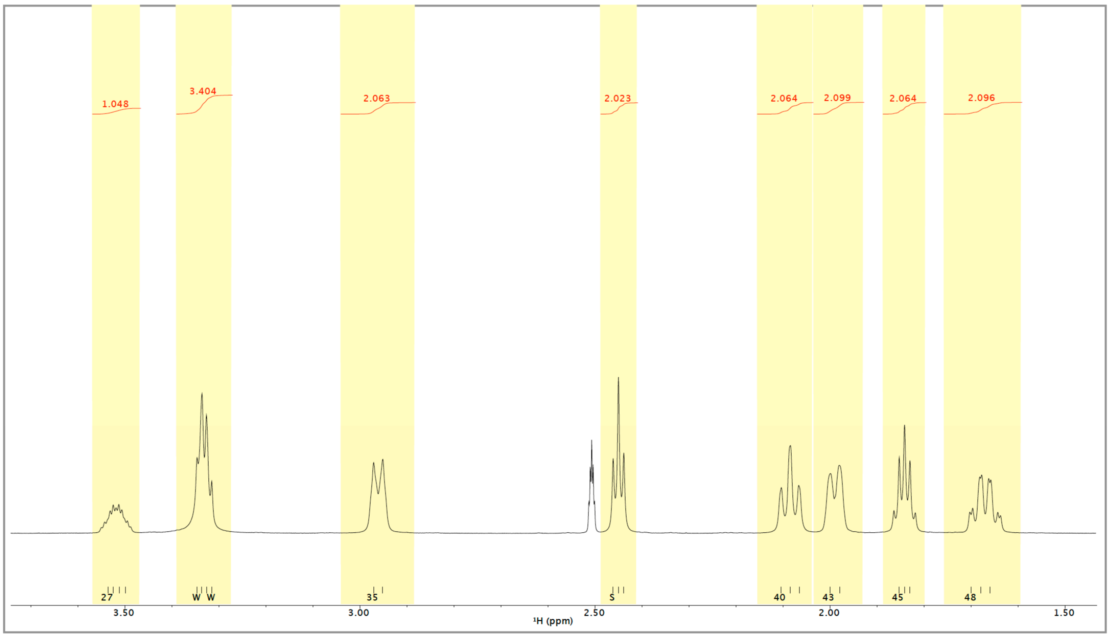

Figure C12: ^{1}H NMR spectrum of compound **6** in DMSO-d6, aliphatic region.

References

Abel, M., Luu, H., Micetich, R., Nguyen, D., Oreski, A., Tempest, M., & Daneshtalab, M. (1996). Synthesis of azolylalkylquinolines with cytotoxic activity. *Journal of Heterocyclic Chemistry* ***33***(2), 415-420.

Allais, A., Meier, J. (1969). Neue Chinolinderivate und Verfahren zu ihrer Herstellung. German Patent, DE1815467.

Andersag, H. (1948). Antimalariamittel aus der Gruppe halogensubstitutierter Chinolinverbindungen. *Chemische Berichte* **81**, 499-507.

Andrews, S.; Burgess, S.J.; Skaalrud, D.; Kelly, J.X.; Peyton, D.H. (2010). Reversal agent and linker variants of reversed chloroquines: activities against Plasmodium falciparum. *Journal of Medicinal Chemistry* **53**, 916-919.

Burgess, S.J., Selzer, A., Kelly, J.X., Smilkstein, M.J., Riscoe, M.K., Peyton, D.H. (2006). A chloroquine-like molecule designed to reverse resistance in Plasmodium falciparum. *Journal of Medicinal Chemistry* **49**, 5623-5625.

Burgess, S.J., Kelly, J.X., Shomloo, S., Wittlin, S., Brun, R., Liebmann, K., Peyton, D.H. (2010). Synthesis, structure-activity relationship, and mode-of-action studies of antimalarial reversed chloroquine compounds. *Journal of Medicinal Chemistry* **52**, 6477-6489.

Claisen, R.L. (1897). Untersuchungen über die Oxymethylenverbindungen. *Justus Liebigs Annalen der Chemie* **297**, 1-98 (the relevant synthesis is on pages 77-8).

De, D., Krogstad, F.M., Byers, L.B., Krogstad, D.J. (1998). Structure activity relationships for antiplasmodial activity among 7-substituted 4-aminoquinolines. *Journal of Medicinal Chemistry* **41**(25), 4918-4926.

Ghosh, B., Antonio, T., Zhen, J., Kharkar, P., Reith, M., & Dutta, A. (2010). Development of (S)-N-6-(2-(4-(Isoquinolin-1-yl)piperazin-1-yl)ethyl)-N-6-propyl-4,5,6, 7-tetrahydrobenzo[d]-thiazole-2,6-diamine and Its Analogue as a D3 Receptor Preferring Agonist: Potent in Vivo Activity in Parkinson's Disease Animal Models. *Journal Of Medicinal Chemistry* **53**(3), 1023-1037.

Gould, R.G., Jr., Jacobs, W.A. (1939). The synthesis of certain substituted quinolines and 5,6-benzoquinolines. *Journal of the American Chemical Society* **61**, 2890-2895.

Hauser, C.R., Reynolds, G.A. (1950). Relative ease of cyclization of 2-, 3-, and 4-aminopyridine derivatives. Synthesis of naphthyridines. *Journal of Organic Chemistry* **15**, 1224-1232.

Iwasaki, N., Sakaguchi, J., Ohashi, T., Takahara, E., Ogawa, N., Yasuda, S., Koshinaka, E., Kato, H., Ito, Y., Sawanishi, H. (1994). Amphoteric Drugs. I. Synthesis and antiallergic activity of [4-(diphenylmethoxy)piperidino)alkanoic acid derivatives. *Chemical and Pharmaceutical Bulletin* **42**(11), 2276-2284.

Price, C.C., Roberts, R.M. (1946). The synthesis of 4-hydroxyquinolines. I. Through ethoxymethylenemalonic ester. *Journal of the American Chemical Society* **68**, 1204-8.

Rhône-Poulenc (1962). Nouveaux dérivés de la quinoléine et leur préparation. Belgian Patent, BE612207.

Surrey, A.R., Cutler, R.A. (1951). The role of phenol in the reaction of 4,7-dichloroquinoline with novol diamine. *Journal of the American Chemical Society* **73**, 2623-6.
